# Circadian Factor BMAL1 in Histaminergic Neurons Regulates Sleep Architecture

**DOI:** 10.1016/j.cub.2014.10.019

**Published:** 2014-12-01

**Authors:** Xiao Yu, Anna Zecharia, Zhe Zhang, Qianzi Yang, Raquel Yustos, Polona Jager, Alexei L. Vyssotski, Elizabeth S. Maywood, Johanna E. Chesham, Ying Ma, Stephen G. Brickley, Michael H. Hastings, Nicholas P. Franks, William Wisden

**Affiliations:** 1 Department of Life Sciences, Imperial College London, Sir Ernst Chain Building, Exhibition Road, London SW7 2AZ, UK; 2 Institute of Neuroinformatics, University of Zurich and ETH Zurich, Winterhurerstrasse 190, Zurich 8057, Switzerland; 3 Neurobiology Division, Medical Research Council Laboratory of Molecular Biology, Cambridge Biomedical Campus, Francis Crick Avenue, Cambridge CB2 0QH, UK

## Abstract

Circadian clocks allow anticipation of daily environmental changes [1]. The suprachiasmatic nucleus (SCN) houses the master clock, but clocks are also widely expressed elsewhere in the body [1]. Although some peripheral clocks have established roles [1], it is unclear what local brain clocks do [2, 3]. We tested the contribution of one putative local clock in mouse histaminergic neurons in the tuberomamillary nucleus to the regulation of the sleep-wake cycle. Histaminergic neurons are silent during sleep, and start firing after wake onset [4, 5, 6]; the released histamine, made by the enzyme histidine decarboxylase (HDC), enhances wakefulness [7, 8, 9, 10, 11]. We found that *hdc* gene expression varies with time of day. Selectively deleting the *Bmal1* (also known as *Arntl* or *Mop3* [12]) clock gene from histaminergic cells removes this variation, producing higher HDC expression and brain histamine levels during the day. The consequences include more fragmented sleep, prolonged wake at night, shallower sleep depth (lower nonrapid eye movement [NREM] δ power), increased NREM-to-REM transitions, hindered recovery sleep after sleep deprivation, and impaired memory. Removing BMAL1 from histaminergic neurons does not, however, affect circadian rhythms. We propose that for mammals with polyphasic/nonwake consolidating sleep, the local BMAL1-dependent clock directs appropriately timed declines and increases in histamine biosynthesis to produce an appropriate balance of wake and sleep within the overall daily cycle of rest and activity specified by the SCN.

## Highlights

• The first role for a putative local clock in sleep regulation is identified
• Local expression of BMAL1 directs rhythmic synthesis of histamine
• Local BMAL1 regulates balance of sleep-wake activity within the circadian cycle
• Local BMAL1 specifies sufficient spontaneous and recovery sleep during the circadian day

## Teaser

The suprachiasmatic nucleus houses the master circadian clock, but local clocks are also found in many cells. It is unclear what local brain clocks do. Yu et al. reveal that a local clock in histaminergic neurons determines fluctuations of the “wakefulness transmitter” histamine, thus balancing sleep and wake within the animal’s circadian behavior.

## Results and Discussion

### A Putative BMAL1-Driven Clock in Histaminergic Neurons

**Figure 1:**
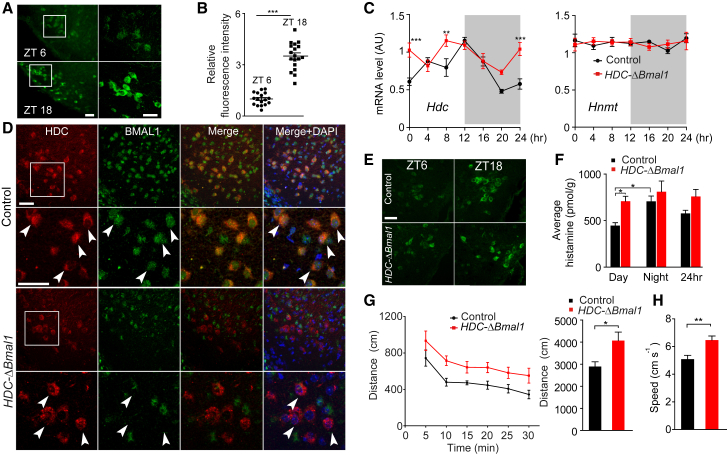
Rhythmic Expression of HDC and Histamine Requires BMAL1 in HDC-Positive Cells (A and B) Expression of HDC in the mouse TMN varies with time of day. The intensity of immunocytochemical staining of neurons with antiserum to HDC (green) was higher at ZT18 than at ZT6 (n = 3 mice in both groups; n = 17 cells in control mice; n = 18 cells in *HDC-ΔBmal1* mice). Boxed regions in (A) are shown at higher power. The scale bars represent 40 μm. The graph (B) was obtained by imaging the fluorescence intensity of individual neurons; bars indicate SEM; ^∗∗∗^p < 0.001. (C) Quantitative PCR analysis of RNA from posterior hypothalamic tissue shows that transcripts encoding HDC vary with time of day, but in *HDC-ΔBmal1* mice (red traces) these rhythms were altered. HNMT transcript levels were unchanged; all transcript levels were normalized to expression of the *18S rRNA* gene. Bars represent SEM; ^∗∗^p < 0.01, ^∗∗∗^p < 0.001. (D) Histaminergic neurons in the TMN area, identified by immunocytochemistry with HDC (red), also contained BMAL1 protein (green); in *HDC-ΔBmal1* mice, BMAL1 staining was selectively removed. Magnifications are shown in the boxed regions. 4′,6-diamidino-2-phenylindole (DAPI) (blue) shows the position of cell nuclei. Arrowheads indicate examples of HDC-positive cells with BMAL1 (control) or without BMAL1 (*HDC-ΔBmal1* knockouts). The scale bars represent 40 μm. (E) Compared with littermate controls, HDC protein is elevated at all ZT points in the TMN of *HDC-ΔBmal1* mice. The scale bar represents 40 μm. (F) Average histamine levels are elevated in *HDC-ΔBmal1* brains during the day (bars represent SEM; ^∗^p < 0.05). (G and H) *HDC-ΔBmal1* mice (red trace) traveled farther than littermate control mice in a 30 min period (G), and speed in total 30 min was higher (H) (n = 10 control; n = 10 *HDC-ΔBmal1*; bars represent SEM; ^∗^p < 0.05, ^∗∗^p < 0.01). See also Figures S1–S3.

Tuberomamillary nucleus (TMN) neurons expressing the *histidine decarboxylase* (*hdc*) gene are the sole neuronal source of histamine [13, 14, 15]. The *hdc* gene shows haploinsufficiency: a 2-fold decrease in *hdc* mRNA levels halves the brain content of histamine in mice [16, 17], and in humans, having only one functional *hdc* allele produces a type of Tourette syndrome [16]. Thus, modest changes in *hdc* transcript levels in TMN neurons can change the amount of histamine released and influence behavior. Changes in *hdc* mRNA levels also seem to occur in the normal daily cycle. *hdc* mRNA levels in human hypothalamus are 1.6-fold higher for daytime deaths [18], and HDC enzyme activity and histamine levels in rat brain vary with time of day [19, 20, 21]. In agreement with these data, immunocytochemical staining for HDC protein in mouse TMN neurons was stronger at zeitgeber time (ZT)18 (night, mid-lights off, the period when the animals are more active) than at ZT6 (day, mid-lights on) (3.5 ± 0.19 versus 1 ± 0.09 arbitrary units [AUs]; unpaired two-tailed t test, p < 0.001) (Figures 1A and 1B). In control mice there was also a 1.5-fold variation in *hdc* transcript levels over 24 hr (unpaired two-tailed, t test, p < 0.05): *hdc* mRNA was highest at the start of the night (ZT12), declined during the night, and increased during the day (Figure 1C). By contrast, transcripts encoding the enzyme that inactivates histamine, histamine N-methyltransferase (HNMT), did not show daily variation in the TMN area (Figure 1C). This daily variation in HDC expression could indicate a clock-like mechanism in histaminergic neurons. Indeed, histaminergic neurons express the core clock protein BMAL1 (Figure 1D). (BMAL1 antisera specificity was confirmed by the absence of staining in suprachiasmatic nucleus [SCN] sections from *BMAL1* global knockout brains [Figure S1A available online].)

### Removing BMAL1 from Histaminergic Neurons Changes the Local Expression of Core Circadian Genes and Elevates *hdc* Expression

We crossed *HDC-Cre* mice [22] with animals containing a floxed *Bmal1* gene [23] (Figure S1B). The resulting *HDC-ΔBmal1* mice were similar to littermate controls in weight (control weight, 26.6 ± 0.6 g, n = 5; *HDC-ΔBmal1* weight, 27 ± 0.7 g, n = 5; unpaired two-tailed t test, p *=* 0.34) and seemed healthy. All the HDC-positive cells lost BMAL1 (Figure 1D). In our characterization of the *HDC-Cre* mice, we found that transient developmental expression of the *hdc* gene produced recombination in several additional places, in particular the dorsal lateral geniculate (DLG) thalamic nucleus, the ventral medial (VM) hypothalamic nucleus, and Purkinje neurons [22]. By immunostaining, there was no indication of BMAL1 loss from the DLG, VMH, and cerebellum of *HDC-ΔBmal1* brains (Figures S1C–S1E); *Bmal1* and *per1* transcript levels in these regions were also unchanged (two-way ANOVA and post hoc Bonferroni, p > 0.05) (Figure S1F). The failure to delete the *bmal1* gene in these areas likely reflects that the particular floxed allele is relatively Cre insensitive, requiring sustained doses of Cre to produce recombination [24].

BMAL1 could serve housekeeping functions unrelated to its clock role. To see whether removing BMAL1 from histaminergic neurons disrupted the local clock, we examined the expression of core clockwork-associated genes in the TMN of control and *HDC-ΔBmal1* mice. In littermate control mice, *Per1*, *Cry1*, and *Rev-erbα* mRNA levels peaked around the beginning of the night (Figure S1G); in *HDC-ΔBmal1* mice, the expression rhythms of these three genes across the light-dark cycle were flattened; *Per1* and *Cry1* mRNA levels were, on average, higher, whereas *Rev-erbα* levels were significantly lower (Figure S1G) (two-way ANOVA and post hoc Bonferroni, ^∗^p < 0.05, ^∗∗^p < 0.01; Cosinor analysis [cosinor.exe, version 2.3; http://www.circadian.org/softwar.html]; *Per1*: control: amplitude, 0.63, p < 0.05; *HDC-ΔBmal1*: p *=* 0.27; *Cry1*: control: amplitude, 0.25, p < 0.05; *HDC-ΔBmal1*: p = 0.25; *Rev-erbα*: control: amplitude, 0.9, p = 0.01; *HDC-ΔBmal1*: amplitude, 0.29, p = 0.05; Cosinor p values are related to comparisons of goodness of cosine fit). Furthermore, the rhythmic expression of PER2 protein was abolished in histaminergic neurons in *HDC-ΔBmal1* mice (Figure S1H; the specificity of the PER2 antiserum was confirmed in *per2* knockout mice [25]). These results indicate that BMAL1 deletion from histaminergic neurons has likely disrupted their local clock mechanism.

In the *HDC-ΔBmal1* mice, *hdc* gene expression showed a disrupted 24 hr pattern (two-way ANOVA and post hoc Bonferroni, ^∗∗^p < 0.01, ^∗∗∗^p < 0.001), and *hdc* transcript levels and protein were overall higher in the day and the late night. This produced increased brain histamine levels in the day (Figure 1F; two-way ANOVA or one-way ANOVA and post hoc Bonferroni, ^∗^p < 0.05). To test the behavioral consequence of upregulated *hdc* gene expression in TMN neurons, we examined locomotor activity in an open field. *HDC-ΔBmal1* mice traveled farther and at higher speeds (Figures 1G and 1H) than littermate controls (unpaired two-tailed t test, ^∗^p < 0.05, ^∗∗^p < 0.01).

BMAL1-CLOCK dimers can either activate or repress target genes [26, 27]. Is the *hdc* gene directly repressed by BMAL1? The 5′ region of the mouse *hdc* gene contains an E box. BMAL1-CLOCK dose-dependently increased *hdc promoter*-*luciferase* gene expression (Figure S2A) (one-way ANOVA and post hoc Bonferroni, ^∗∗∗^p < 0.001), but not when the E box was mutated (Figure S2B). This was the opposite of the in vivo situation, when *hdc* transcript levels increased after BMAL1 deletion. Thus, in histaminergic neurons, BMAL1 could recruit a repressor complex onto the *hdc* promoter [27]. Alternatively, *RORE* sequences in the *hdc* gene could bind the repressor and core clock protein REV-ERBα [28, 29]. Diminished REV-ERBα levels in the TMN of *HDC-ΔBmal1* mice (Figure S1G) might derepress the *hdc* gene.

### Intrinsic Electrical Properties of Histaminergic Neurons Are Not Influenced by BMAL1

SCN neurons show cell-intrinsic circadian regulation of their electrophysiological parameters, partly determining when these neurons fire [30, 31, 32]. We made whole-cell current-clamp recordings of histaminergic neurons from littermate and *HDC-ΔBmal1* mice during night and day (Figure S3C). Resting membrane potential, input conductance, current injection to threshold of action potential firing, capacitance, and membrane time constant were unaffected by time of day or the absence of BMAL1 (Figure S3C). We expect that *HDC-ΔBmal1* histaminergic neurons will fire action potentials normally but release more histamine.

### *HDC-ΔBmal1* Mice Have an Unchanged Circadian Wheel-Running Behavior

**Figure 2:**
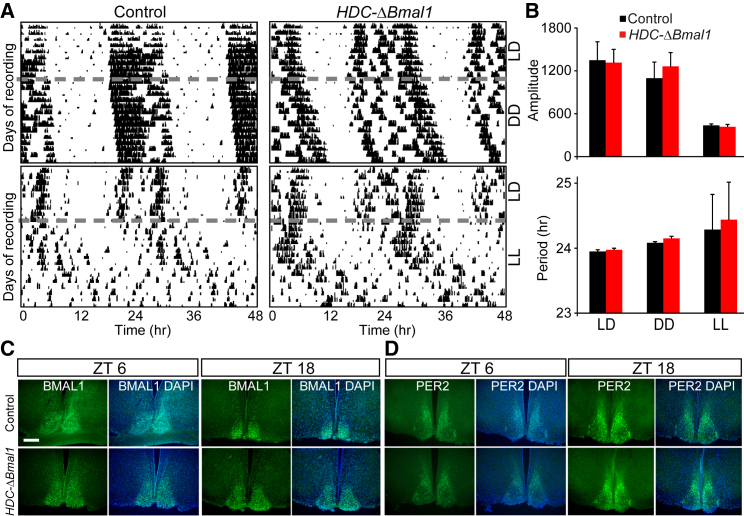
*HDC-ΔBmal1* Mice Have a Functionally Normal Circadian Clock (A) Representative wheel-running actograms from homozygous *loxBmal1* mice and *HDC-ΔBmal1* mice. Mice were initially entrained to 12 h white light, 12 h dim red light (LD) and then transferred to continuous dim red light (DD) or continuous white light (LL). (B) *HDC-ΔBmal1* mice (n = 6) and littermate controls (n = 8) did not have differing circadian periods or amplitudes during LD, LL, or DD (bars represent SEM; p > 0.05). (C and D) Immunocytochemical analysis shows that the circadian rhythm of BMAL1 (green) and PER2 (green) expression is unaffected in the SCN of *HDC-ΔBmal1* mice. Sections are counterstained to show all cell nuclei with DAPI. The scale bar represents 0.5 mm.

*HDC-ΔBmal1* knockout mice had an unchanged behavioral circadian rhythm and phase, compared with littermate controls, as assessed by wheel running in free-running conditions of constant darkness (DD) (unpaired two-tailed t test, p > 0.05) (Figures 2A and 2B) [25]. In free-running constant light (LL), both genotypes were more variable in period length than in LD or DD (Figure 2A). However, the amplitude of the peak period was lower and more variable in LL than in LD and DD, indicating the mice were equally less active in LL than in LD or DD, regardless of genotype (Figures 2A and 2B). Within the SCN, the circadian variation in BMAL1 and PER2 proteins was unchanged between *HDC-ΔBmal1* knockout mice and littermate controls (Figures 2C and 2D); there was little variation in BMAL1 staining intensity in the SCN between ZT6 and ZT18 (Figure 2C), highlighting that although BMAL1 is the core component of the clock, its levels change little during the circadian cycle. CLOCK and BMAL1 are often constitutively bound to E boxes. The critical rhythm for BMAL1-CLOCK activity arises from PER-CRY, which arrives to inhibit, and then dissociates from, the BMAL1-CLOCK complex [33]. PER2 staining in the SCN of both groups of mice increased at ZT18 compared with ZT6 (Figure 2D). Thus, the *HDC-ΔBmal* mice had an unaffected SCN molecular clock and circadian pace making.

### *HDC-ΔBmal1* Mice Have More Fragmented Sleep

**Figure 3:**
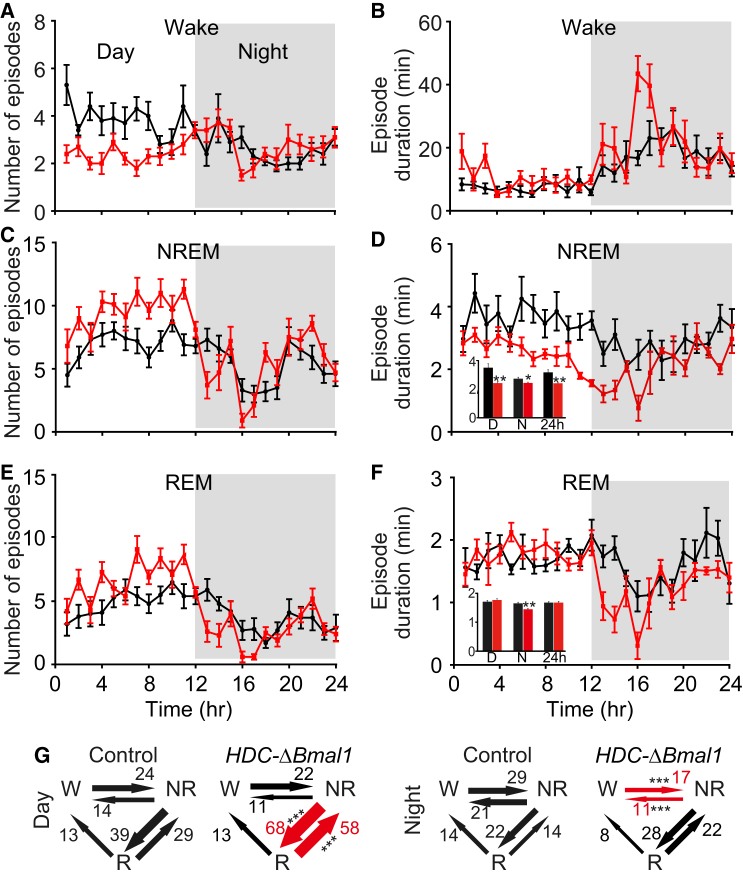
*HDC-ΔBmal1* Mice Have Fragmented Sleep (A–F) The number of vigilance (wake, NREM, and REM) episodes (A, C, and E, respectively) and episode duration (B, D, and F, respectively) over the 24 hr interval for *HDC-ΔBmal1* mice (n = 10) (red traces) and littermate controls (n = 10). Bars represent SEM; ^∗^p < 0.05, ^∗∗^p < 0.01. D, day; N, night. (G) Number of transitions between wake (W), NREM (NR), and REM (R) sleep in the day and night. Significant differences in transition numbers are shown with red arrows; ^∗∗∗^p < 0.001. See also Figure S4.

Mice unable to synthesize histamine (*HDC* knockouts) show normal sleep-wake behavior throughout most of the 24 hr cycle, except they are significantly less awake just before, and for the first few hours after, the start of the night [10]. It is intriguing that *HDC* knockout mice have a selective deficit in *anticipating* lights off, further suggesting a circadian involvement of histaminergic neurons. In contrast to *HDC* knockout mice, the *HDC-ΔBmal1* mice have a gain of function in the histaminergic system. We looked at the consequences for the sleep-wake cycle (Figure 3; Figure S4). Sleep experiments and nontethered electroencephalogram (EEG) analysis were performed using Neurologger2 devices [22, 34]. For the first part of the night, *HDC-ΔBmal1* mice were more awake, as assessed by electromyogram (EMG) and the ratio of δ:θ power in the EEG, than littermate controls (Figures S4A–S4C), namely the opposite of *HDC* knockout mice [10]. As the night progressed, the EEG:EMG ratios of the *HDC-ΔBmal1* mice became similar to littermate controls (Figure S4A). Some the *HDC-ΔBmal1* mice had long (up to 40 min) periods of uninterrupted waking (Figure 3B). The total wake time, however, of *HDC-ΔBmal1* mice averaged over 24 hr was unchanged (693 ± 21 min versus 693 ± 12 min, unpaired two-tailed t test, p > 0.05), but over the night they spent more time awake than littermate control mice and less time awake during the day (night: 420 ± 16 min versus 461 ± 10 min, unpaired two-tailed t test, p < 0.05; day: 273 ± 9 min versus 231 ± 7 min, unpaired two-tailed t test, p < 0.05) (Figure S4A). Throughout the 24 hr, during the wake periods, the *HDC-ΔBmal1* mice had higher θ frequencies in the EEG than littermate controls (two-way ANOVA and post hoc Bonferroni, p < 0.05) (Figure S4D).

The amount of nonrapid eye movement (NREM) sleep was similar between *HDC-ΔBmal1* and control mice (Figure S4B) (488 ± 11 min versus 427 ± 20 min, unpaired two-tailed t test, p > 0.05), but NREM power was lower (Figure S4E; see next section) (two-way ANOVA and post hoc Bonferroni, ^∗^p < 0.05). During the day, *HDC-ΔBmal1* mice had more NREM episodes than controls (Figure 3C), but these episodes were shorter (Figure 3D) (3.5 ± 0.3 min versus 2.4 ± 0.3 min, unpaired two-tailed t test, ^∗∗^p < 0.01). The amount of REM sleep in *HDC-ΔBmal1* mice compared with littermate controls was higher in the day (Figure S4C): there were more episodes (Figure 3E), although episode duration was unchanged (Figure 3F) (1.7 ± 0.04 min versus 1.8 ± 0.04 min, unpaired two-tailed t test, p > 0.05); however, REM episode duration was shorter in the *HDC-ΔBmal1* mice (Figure 3F) during the night (1.6 ± 0.04 min versus 1.4 ± 0.03 min, unpaired two-tailed t test, ^∗∗^p < 0.01).

The daytime sleep architecture of *HDC-ΔBmal1* mice differed from littermate control mice (Figure 3G). *HDC-ΔBmal1* mice had more “NREM-to-REM” (39 ± 2 versus 68 ± 2, unpaired two-tailed t test, ^∗∗∗^p < 0.001) and “REM-to-NREM” (29 ± 1 versus 58 ± 2, unpaired two-tailed t test, ^∗∗∗^p < 0.001) transitions during the day (Figure 3G). During the night, there was no difference between genotypes in NREM-REM transitions (22 ± 3 versus 28 ± 2, unpaired two-tailed t test, p > 0.05) (Figure 3G); however, *HDC-ΔBmal1* mice had fewer wake-to-NREM transitions (29 ± 3 versus 17 ± 1, unpaired two-tailed t test, ^∗∗∗^p < 0.001) and vice versa (21 ± 3 versus 11 ± 1, unpaired two-tailed t test, ^∗∗∗^p < 0.001) (Figure 3G), reflecting that they were awake more (Figure 3B). Thus, sustained elevated histamine in *HDC-ΔBmal1* mice changed the sleep-wake architecture.

### After Sleep Deprivation, the Recovery Sleep of *HDC-ΔBmal1* Mice Is Shorter with Less Power

**Figure 4:**
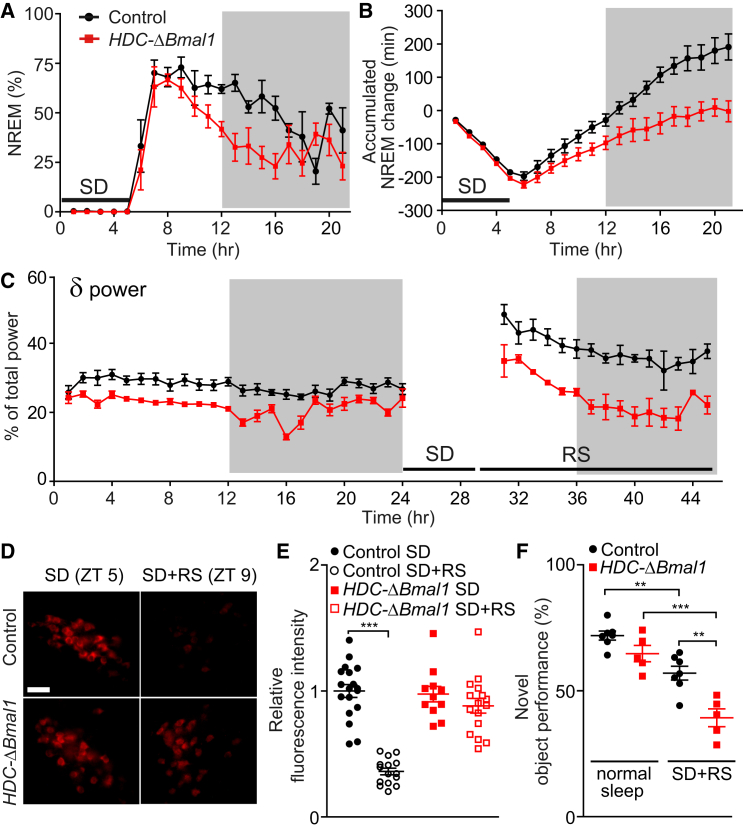
*HDC-ΔBmal1* Mice Have Compromised Recovery Sleep and Elevated HDC Expression after Sleep Deprivation and Compromised Novel Object Recognition (A) After 5 hr of sleep deprivation (SD) during the day, littermate control mice (n = 5) had sustained NREM sleep; in contrast, the recovery sleep time of the *HDC-ΔBmal1* mice (red trace; n = 5) was reduced. (B) Rate of accumulated NREM change following SD. (C) Graph of NREM δ power of *HDC-ΔBmal1* mice and littermate controls before and after sleep deprivation. RS, recovery sleep. (D) HDC immunocytochemical staining in littermate control and *HDC-ΔBmal1* mice at the end of SD (ZT5) and following 4 hr of recovery sleep (ZT9). The scale bar represents 40 μm. (E) Quantification of relative HDC expression following sleep deprivation and recovery sleep. Each point represents an HDC-positive neuron. Bars represent SEM; ^∗∗∗^p < 0.001. (F) Control littermates (n = 7) and *HDC-ΔBmal1* mice (n = 5) were trained for 10 min to explore the same object, and then the mice were allowed 22 hr of normal sleep-wake behavior or subjected to 5 hr of sleep deprivation and allowed 17 hr of recovery sleep. One of the objects was then exchanged with a new object, and the mice were again tested for 10 min. Both control littermates and *HDC-ΔBmal1* mice performed worse after SD + RS, compared with those that had only a normal sleep-wake experience; however, *HDC-ΔBmal1* mice performed less well. The “novel object performance” was defined as the time exploring the novel object divided by the time exploring the familiar object and novel object, expressed as a percentage. Bars represent SEM; ^∗∗^p < 0.01, ^∗∗∗^p < 0.001. See also Figure S4.

*HDC-ΔBmal1* mice and littermate controls were sleep deprived for 5 hr during the start of the day [35]. Mice were placed into a novel cage, and objects (plastic tubes, pieces of paper) were introduced that were exchanged each hour. This method reliably prevented sleep (Figure 4A). Consistent with the raised brain histamine levels in *HDC-ΔBmal1* mice, the EEG profiles between the genotypes differed during sleep deprivation: littermate control mice had frequencies distributed in the δ-to-θ range (2–10 Hz), with two peaks at 2 and 8 Hz, but the *HDC-ΔBmal1* mice had a single broad peak of enhanced power relative to controls, centered in the θ range (Figures S4G and S4H).

After sleep deprivation, mice slept freely in their home cages. Littermate control mice had a recovery sleep lasting about 10–12 hr (Figure 4A), with sustained NREM periods remaining 6 hr into the night. They reaccumulated their NREM sleep at a rate of approximately 30 min extra NREM sleep per hour (Figure 4B). The δ power in the EEG of littermate control mice remained elevated, compared with presleep deprivation levels, for some 12 hr after sleep deprivation (Figure 4C). By contrast, *HDC-ΔBmal1* mice did not sustain their recovery sleep: it was about 6 hr shorter than sleep-deprived control littermates (Figure 4A), and their enhanced δ power of recovery sleep, already lower compared with littermate controls before sleep deprivation, remained lower as it declined to baseline (Figure 4C; Figure S4H). *HDC-ΔBmal1* mice reaccumulated their NREM sleep at a slower rate than control mice: 12.5 min extra NREM sleep per hour (Figure 4B). Because *HDC-ΔBmal1* mice had more REM baseline sleep than littermate controls during the day (Figure 3G), they had more REM loss during sleep deprivation, namely they had more REM sleep to lose by sleep deprivation (Figure S4J). *HDC-ΔBmal1* mice also had a quicker reaccumulation of REM sleep during recovery sleep (Figure S4J). In the recovery stage after sleep deprivation, *HDC-ΔBmal1* mice had more transitions from NREM to REM, which caused more REM gain but less NREM gain. The reason could be that *hdc* expression was stronger in the *HDC-ΔBmal1* mice during recovery sleep (see next section).

### HDC Expression Is Reversibly Elevated by Sleep Deprivation and Requires BMAL1 to Reduce Its Expression to Baseline

We examined HDC expression in TMN neurons at the end of the sleep deprivation period (ZT5; 5 hr of sleep deprivation). ZT5 is when HDC and histamine levels are normally lower (Figure 1). At the end of the deprivation period, however, HDC expression was at the higher nighttime levels in both littermate controls and *HDC-ΔBmal1* mice (Figure 4D), suggesting that sleep deprivation increases *hdc* gene expression. Consistently, sleep deprivation raises histamine levels in cerebrospinal fluid [36]. In control mice, 4 hr into recovery sleep, HDC protein expression had decreased (roughly halved) to typical ZT6 levels (1% ± 0.05% versus 0.36% ± 0.03%, AUs, one-way ANOVA and post hoc Bonferroni, ^∗∗∗^p < 0.001) (Figure 4E). In *HDC-ΔBmal1* mice, HDC protein expression remained elevated (Figure 4E). Presumably, in wild-type mice, *hdc* expression increases to combat the effects of sleep deprivation, and BMAL1 represses *hdc* gene expression back to baseline levels, ensuring good recovery sleep. Without BMAL1 in histaminergic neurons, the *hdc* gene expression level stayed flat and higher because it was already high before sleep deprivation and could not be further induced.

### *HDC-ΔBmal1* Mice Are More Impaired in Novel Object Recognition following Sleep Deprivation and Recovery Sleep

We investigated whether the diminished recovery sleep after sleep deprivation of *HDC-ΔBmal1* mice affected their ability at novel object recognition (Figure 4F). In mice, this memory task is sensitive to sleep deprivation [35, 37]. Control littermates and *HDC-ΔBmal1* mice were tested [35] either during the night phase of their normal sleep-wake cycle or after 5 hr of sleep deprivation followed by 17 hr of recovery sleep. Mice were trained for 10 min in the open field with the same objects; control littermates and *HDC-ΔBmal1* mice spent equal time exploring the two objects. In normal sleep-wake cycle conditions, control littermates and *HDC-ΔBmal1* mice performed the same (72% ± 2% versus 65% ± 3%, one-way ANOVA and post hoc Bonferroni, p > 0.05) (Figure 4F). For both genotypes, sleep deprivation impaired performance in recognizing the novel object, even after 17 hr of recovery sleep (Figure 4F); however, *HDC-ΔBmal1* mice performed worse (56% ± 3% versus 39% ± 3%, one-way ANOVA and post hoc Bonferroni, ^∗∗^p < 0.01) (Figure 4F). Thus, the reduced recovery of NREM sleep in *HDC-ΔBmal1* mice, compared to littermate controls, impaired cognitive function (Figures 4A and 4B).

### Conclusions

Circadian transcription factors regulate arousal and sleep [3, 12, 38, 39]. Our work reveals a specified function for local clock factors in histaminergic circuitry controlling arousal. BMAL1 in histaminergic neurons promotes a daily 1.5-fold fluctuation in *hdc* gene expression, with lower mRNA levels during the day. We propose that the local BMAL1-dependent clock mechanism suppresses daytime histaminergic tone and thereby facilitates appropriately timed intervals of sleep and wake synchronized to the animal’s overall circadian behavior.

## Supplemental Information

Document S1. Supplemental Experimental Procedures and Figures S1–S4

Document S2. Article plus Supplemental Information

## References

[1] Mohawk J.A., Green C.B., Takahashi J.S. (2012). Central and peripheral circadian clocks in mammals. Annu. Rev. Neurosci..

[2] Kyriacou C.P., Hastings M.H. (2010). Circadian clocks: genes, sleep, and cognition. Trends Cogn. Sci..

[3] Franken P. (2013). A role for clock genes in sleep homeostasis. Curr. Opin. Neurobiol..

[4] Takahashi K., Lin J.S., Sakai K. (2006). Neuronal activity of histaminergic tuberomammillary neurons during wake-sleep states in the mouse. J. Neurosci..

[5] Saper C.B., Fuller P.M., Pedersen N.P., Lu J., Scammell T.E. (2010). Sleep state switching. Neuron.

[6] Lin J.S., Anaclet C., Sergeeva O.A., Haas H.L. (2011). The waking brain: an update. Cell. Mol. Life Sci..

[7] Lin J.S., Sakai K., Jouvet M. (1988). Evidence for histaminergic arousal mechanisms in the hypothalamus of cat. Neuropharmacology.

[8] Haas H., Panula P. (2003). The role of histamine and the tuberomamillary nucleus in the nervous system. Nat. Rev. Neurosci..

[9] Anaclet C., Parmentier R., Ouk K., Guidon G., Buda C., Sastre J.P., Akaoka H., Sergeeva O.A., Yanagisawa M., Ohtsu H. (2009). Orexin/hypocretin and histamine: distinct roles in the control of wakefulness demonstrated using knock-out mouse models. J. Neurosci..

[10] Parmentier R., Ohtsu H., Djebbara-Hannas Z., Valatx J.L., Watanabe T., Lin J.S. (2002). Anatomical, physiological, and pharmacological characteristics of histidine decarboxylase knock-out mice: evidence for the role of brain histamine in behavioral and sleep-wake control. J. Neurosci..

[11] Zant J.C., Rozov S., Wigren H.K., Panula P., Porkka-Heiskanen T. (2012). Histamine release in the basal forebrain mediates cortical activation through cholinergic neurons. J. Neurosci..

[12] Bunger M.K., Wilsbacher L.D., Moran S.M., Clendenin C., Radcliffe L.A., Hogenesch J.B., Simon M.C., Takahashi J.S., Bradfield C.A. (2000). Mop3 is an essential component of the master circadian pacemaker in mammals. Cell.

[13] Watanabe T., Taguchi Y., Hayashi H., Tanaka J., Shiosaka S., Tohyama M., Kubota H., Terano Y., Wada H. (1983). Evidence for the presence of a histaminergic neuron system in the rat brain: an immunohistochemical analysis. Neurosci. Lett..

[14] Panula P., Yang H.Y., Costa E. (1984). Histamine-containing neurons in the rat hypothalamus. Proc. Natl. Acad. Sci. USA.

[15] Bayliss D.A., Wang Y.M., Zahnow C.A., Joseph D.R., Millhorn D.E. (1990). Localization of histidine decarboxylase mRNA in rat brain. Mol. Cell. Neurosci..

[16] Castellan Baldan L., Williams K.A., Gallezot J.D., Pogorelov V., Rapanelli M., Crowley M., Anderson G.M., Loring E., Gorczyca R., Billingslea E. (2014). Histidine decarboxylase deficiency causes Tourette syndrome: parallel findings in humans and mice. Neuron.

[17] Ohtsu H., Tanaka S., Terui T., Hori Y., Makabe-Kobayashi Y., Pejler G., Tchougounova E., Hellman L., Gertsenstein M., Hirasawa N. (2001). Mice lacking histidine decarboxylase exhibit abnormal mast cells. FEBS Lett..

[18] Shan L., Hofman M.A., van Wamelen D.J., Van Someren E.J., Bao A.M., Swaab Dick F. (2012). Diurnal fluctuation in histidine decarboxylase expression, the rate limiting enzyme for histamine production, and its disorder in neurodegenerative diseases. Sleep.

[19] Orr E., Quay W.B. (1975). Hypothalamic 24-hour rhythms in histamine, histidine, decarboxylase and histamine-N-methyltransferase. Endocrinology.

[20] Rozov S.V., Zant J.C., Karlstedt K., Porkka-Heiskanen T., Panula P. (2014). Periodic properties of the histaminergic system of the mouse brain. Eur. J. Neurosci..

[21] Prast H., Dietl H., Philippu A. (1992). Pulsatile release of histamine in the hypothalamus of conscious rats. J. Auton. Nerv. Syst..

[22] Zecharia A.Y., Yu X., Götz T., Ye Z., Carr D.R., Wulff P., Bettler B., Vyssotski A.L., Brickley S.G., Franks N.P., Wisden W. (2012). GABAergic inhibition of histaminergic neurons regulates active waking but not the sleep-wake switch or propofol-induced loss of consciousness. J. Neurosci..

[23] Storch K.F., Paz C., Signorovitch J., Raviola E., Pawlyk B., Li T., Weitz C.J. (2007). Intrinsic circadian clock of the mammalian retina: importance for retinal processing of visual information. Cell.

[24] Husse J., Zhou X., Shostak A., Oster H., Eichele G. (2011). Synaptotagmin10-Cre, a driver to disrupt clock genes in the SCN. J. Biol. Rhythms.

[25] Meng Q.J., Maywood E.S., Bechtold D.A., Lu W.Q., Li J., Gibbs J.E., Dupré S.M., Chesham J.E., Rajamohan F., Knafels J. (2010). Entrainment of disrupted circadian behavior through inhibition of casein kinase 1 (CK1) enzymes. Proc. Natl. Acad. Sci. USA.

[26] Kondratov R.V., Shamanna R.K., Kondratova A.A., Gorbacheva V.Y., Antoch M.P. (2006). Dual role of the CLOCK/BMAL1 circadian complex in transcriptional regulation. FASEB J..

[27] Nguyen K.D., Fentress S.J., Qiu Y., Yun K., Cox J.S., Chawla A. (2013). Circadian gene Bmal1 regulates diurnal oscillations of Ly6C(hi) inflammatory monocytes. Science.

[28] Kojetin D.J., Burris T.P. (2014). REV-ERB and ROR nuclear receptors as drug targets. Nat. Rev. Drug Discov..

[29] Preitner N., Damiola F., Lopez-Molina L., Zakany J., Duboule D., Albrecht U., Schibler U. (2002). The orphan nuclear receptor REV-ERBα controls circadian transcription within the positive limb of the mammalian circadian oscillator. Cell.

[30] Belle M.D., Diekman C.O., Forger D.B., Piggins H.D. (2009). Daily electrical silencing in the mammalian circadian clock. Science.

[31] Colwell C.S. (2011). Linking neural activity and molecular oscillations in the SCN. Nat. Rev. Neurosci..

[32] Granados-Fuentes D., Norris A.J., Carrasquillo Y., Nerbonne J.M., Herzog E.D. (2012). I(A) channels encoded by Kv1.4 and Kv4.2 regulate neuronal firing in the suprachiasmatic nucleus and circadian rhythms in locomotor activity. J. Neurosci..

[33] Forger D.B., Peskin C.S. (2003). A detailed predictive model of the mammalian circadian clock. Proc. Natl. Acad. Sci. USA.

[34] Vyssotski A.L., Dell’Omo G., Dell’Ariccia G., Abramchuk A.N., Serkov A.N., Latanov A.V., Loizzo A., Wolfer D.P., Lipp H.P. (2009). EEG responses to visual landmarks in flying pigeons. Curr. Biol..

[35] Halassa M.M., Florian C., Fellin T., Munoz J.R., Lee S.Y., Abel T., Haydon P.G., Frank M.G. (2009). Astrocytic modulation of sleep homeostasis and cognitive consequences of sleep loss. Neuron.

[36] Soya A., Song Y.H., Kodama T., Honda Y., Fujiki N., Nishino S. (2008). CSF histamine levels in rats reflect the central histamine neurotransmission. Neurosci. Lett..

[37] Palchykova S., Winsky-Sommerer R., Meerlo P., Dürr R., Tobler I. (2006). Sleep deprivation impairs object recognition in mice. Neurobiol. Learn. Mem..

[38] Laposky A., Easton A., Dugovic C., Walisser J., Bradfield C., Turek F. (2005). Deletion of the mammalian circadian clock gene BMAL1/Mop3 alters baseline sleep architecture and the response to sleep deprivation. Sleep.

[39] Naylor E., Bergmann B.M., Krauski K., Zee P.C., Takahashi J.S., Vitaterna M.H., Turek F.W. (2000). The circadian clock mutation alters sleep homeostasis in the mouse. J. Neurosci..

