# Supplementary material for: Circadian Factor BMAL1 in Histaminergic Neurons Regulates Sleep Architecture
Source: Curr Biol. 2014 Dec 1;24(23):2838–44. doi: 10.1016/j.cub.2014.10.019 (PMC4252164; doi:10.1016/j.cub.2014.10.019)
Supplement: Document S1. Supplemental Experimental Procedures and Figures S1–S4 [file mmc1.pdf]

Current Biology, Volume *24*

Supplemental Information

Circadian Factor BMAL1 in Histaminergic Neurons Regulates Sleep Architecture

Xiao Yu, Anna Zecharia, Zhe Zhang, Qianzi Yang, Raquel Yustos, Polona Jager, Alexei L. Vyssotski, Elizabeth S. Maywood, Johanna E. Chesham, Ying Ma, Stephen G. Brickley, Michael H. Hastings, Nicholas P. Franks, and William Wisden

Supplemental Information

Supplemental Figures and Legends

Figure S1

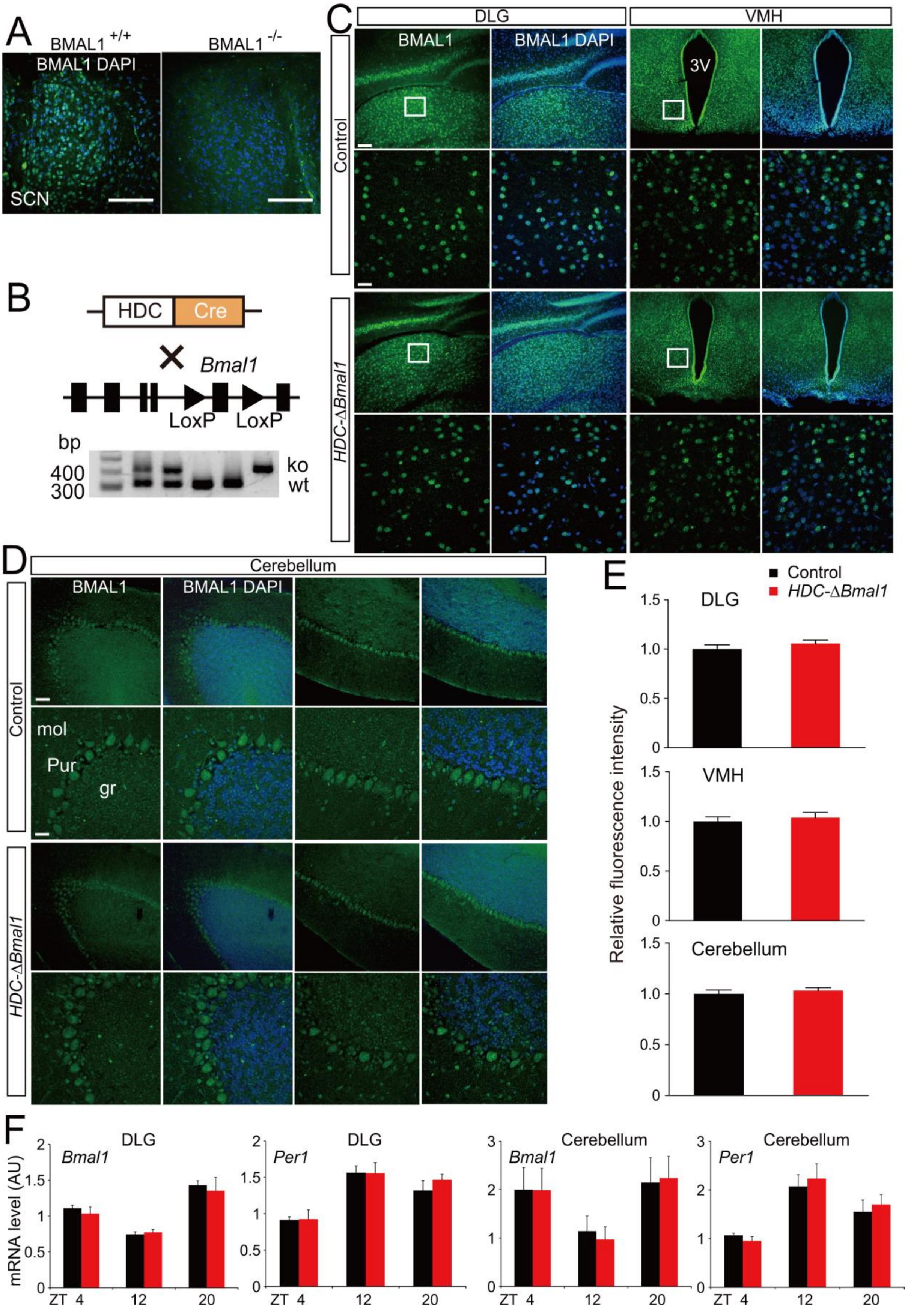
A
BMAL1 +/+
BMAL1 -/-
BMAL1 DAPI
SCN
B
HDC
Cre
Bmal1
LoxP
LoxP
bp
400
300
ko
wt
C
DLG
VMH
BMAL1
BMAL1 DAPI
3V
Control
HDC-ΔBmal1
D
Cerebellum
BMAL1
BMAL1 DAPI
Control
mol
Pur
gr
HDC-ΔBmal1
E
Control
HDC-ΔBmal1
DLG
VMH
Cerebellum
Relative fluorescence intensity
1.5
1.0
0.5
0
F
DLG
Bmal1
Per1
Cerebellum
mRNA level (AU)
ZT 4
12
20

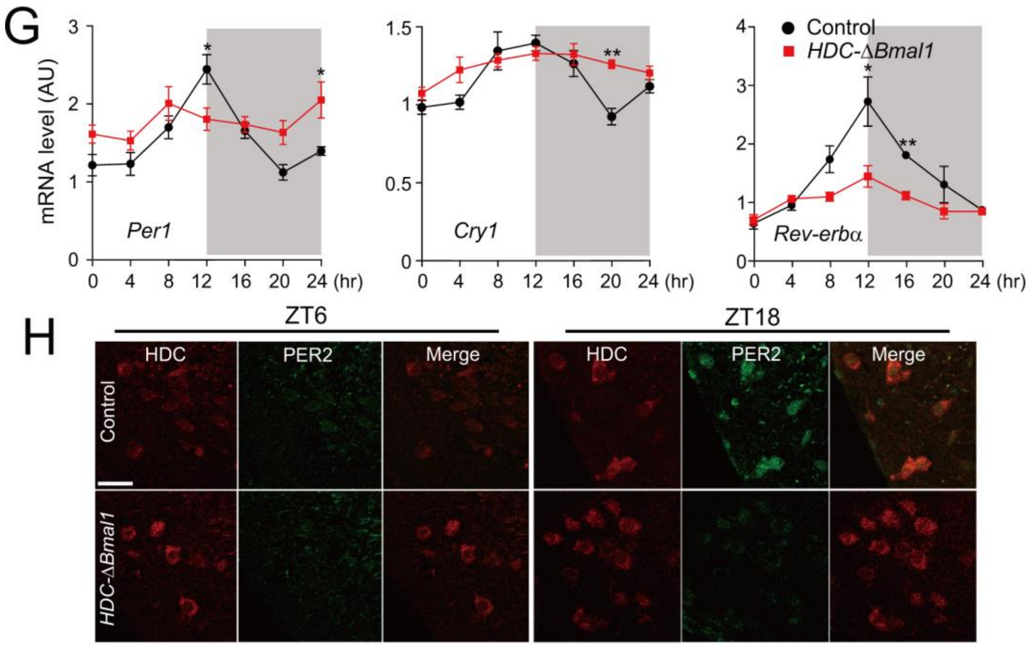

Figure S1. Deletion of BMAL1 from histaminergic neurons disrupts a local clock (related to Figure 1)

(A) To test the specificity of the BMAL1 antibody, SCN brain sections from BMAL1+/+ or BMAL1-/- mice (*loxBmal1* x *Nestin-Cre*) were incubated with the BMAL1 antisera. BMAL1 was detected in control mouse SCN cells but not in BMAL1-/- sections. Scale bar, 100 μm. (B) *HDC-Cre* mediated deletion of the 5th coding exon of the *Bmal1* gene; illustrative PCR genotyping using ear genomic DNA from crosses of *HDC-Cre/loxBmal1* x *loxBmal1/loxBmal1* mice. (C, D) BMAL1 protein expression as seen by immunocytochemistry was unaffected in the dorsal lateral geniculate thalamic (DLG) nucleus, the ventral medial hypothalamus (VMH) (panel C) and the cerebellar Purkinje cells and granule cells (panel D); Pur, Purkinje cells; gr, granule cells; mol, molecular layer. Green images are staining with florescent secondary antibodies to anti-Bmal1 antisera; the blue is a DAPI stain showing all cell nuclei. Scale bars, 0.5 mm (top panel); 20 μm (bottom panel). (E) Flourescence intensity of individual cells was

quantified in DLG (n = 30 control vs. n = 30 *HDC-ΔBmal1*), VMH (n = 40 control vs. n = 40 *HDC-ΔBmal1*) and cerebellum (n = 21 control vs. n = 20 *HDC-ΔBmal1*) using ImageJ; bars represent SEM, $p > 0.05$. (F) Q-PCR analysis of RNA from thalamus tissue (DLG punch) and cerebellum shows that transcripts encoding BMAL1 and PER1 did not show any difference in either genotype, $p > 0.05$). (G) Q-PCR analysis of RNA from posterior hypothalamic tissue (TMN punch) shows that transcripts encoding PER1, CRY1 and REV-ERBα vary with time of day; in *HDC-ΔBmal1* mice (red traces), these rhythms were altered. All transcript levels were normalized to expression of the *18s rRNA* gene. AU, arbitrary units; bars represent SEM, $*p < 0.05$, $**p < 0.01$. (H) In control HDC-positive neurons (staining with HDC antisera shown in red), PER2 immunoreactivity was low at ZT6 and was high at ZT18 (quite similar to the situation in the SCN, see Fig. 2). In HDC neurons in *HDC-ΔBmal1* mice, PER2 staining did not increase at ZT18. Scale bar, 40 μm.

Figure S2.

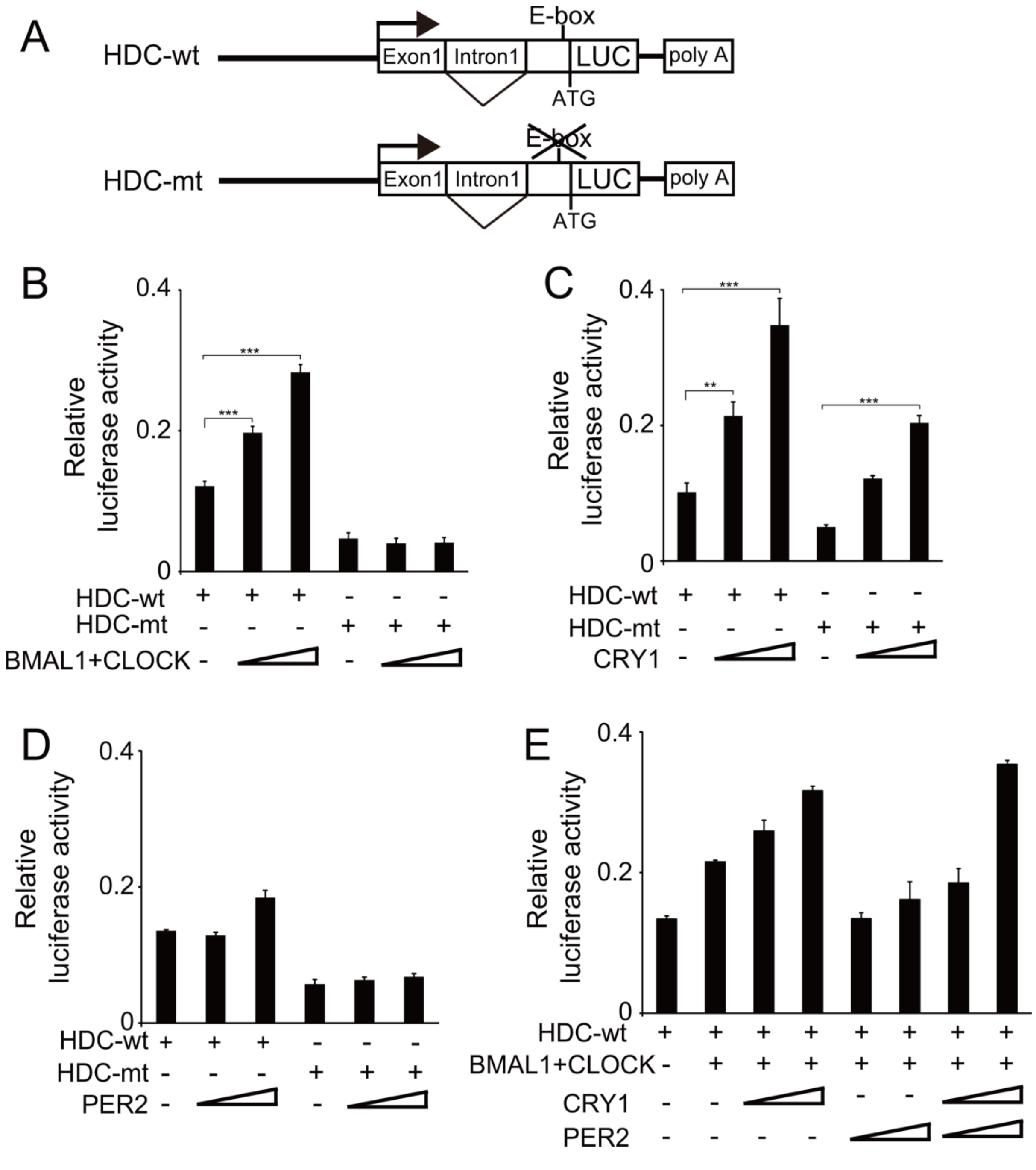

Figure S2. **The mouse *hdc* gene has a canonical E-box in the proximal promoter region which can be activated by BMAL:CLOCK dimers (related to Figure 1).** (A) Transient transfection assays in HEK cells were performed with two luciferase (LUC) reporter gene constructs, containing either the *hdc*

gene proximal promoter with the wild-type (HDC-wt) or mutated E-box (HDC-mt), together with co-transfection of specific combinations of expression plasmids for BMAL1, CLOCK, PER2 and CRY1; (B) The HDC-wt construct had a certain basal activity in HEK cells, even without the addition of exogenous BMAL1:CLOCK; however, this basal expression depended on the E-box, because mutation of the E-box abolished basal expression. Thus presumably the HDC-wt construct is activated to some extent by endogenous BMAL:CLOCK complexes in HEK cells. However, exogenous BMAL1:CLOCK co-transfection increased HDC-wt expression in direct proportion to the amount of BMAL:CLOCK added; in contrast, HDC-mt expression was not increased by BMAL:CLOCK co-transfection, demonstrating the requirement of the E-box (***$p < 0.001$). (C) Because, *cry1* gene expression also increases in *HDC-ΔBmal1* mice (Figure S1G), as found in other BMAL1 deletion studies, we tested if CRY1 protein could activate the *hdc* promoter fragment. CRY1 dose-dependently stimulated *hdc promoter-luciferase* expression, even when the *E*-box was mutated (**$p < 0.01$, ***$p < 0.001$). Thus it could be that the elevated *cry1* gene expression in *HDC-ΔBmal1* neurons drives increased *hdc* gene transcription; (D) the *hdc* proximal promoter activity was unaffected by PER2. (E) CRY1 can still activate the *hdc* promoter even when PER2 is over expressed. Bars represent SEM.

Figure S3.

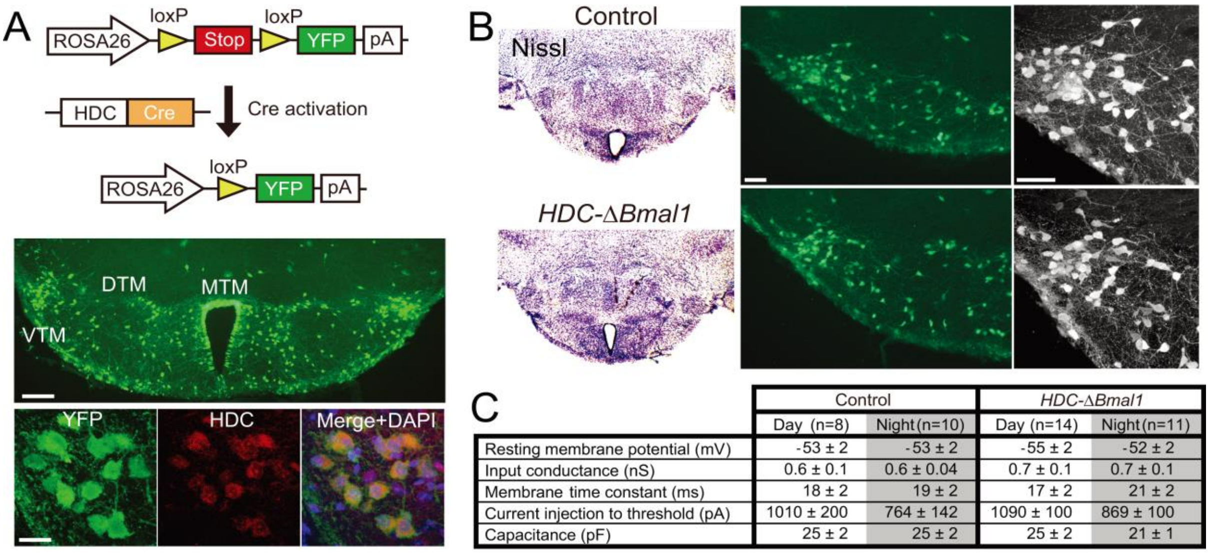

	Control		*HDC-ΔBmal1*	
	Day (n=8)	Night(n=10)	Day (n=14)	Night(n=11)
Resting membrane potential (mV)	-53 ± 2	-53 ± 2	-55 ± 2	-52 ± 2
Input conductance (nS)	0.6 ± 0.1	0.6 ± 0.04	0.7 ± 0.1	0.7 ± 0.1
Membrane time constant (ms)	18 ± 2	19 ± 2	17 ± 2	21 ± 2
Current injection to threshold (pA)	1010 ± 200	764 ± 142	1090 ± 100	869 ± 100
Capacitance (pF)	25 ± 2	25 ± 2	25 ± 2	21 ± 1

Figure S3. Intrinsic electrical properties of histaminergic neurons are not influenced by BMAL1 (related to Figure 1). (A) Histaminergic neurons in the TMN were visualized by crossing *HDC-Cre* mice with mice containing a Rosa26-lox-stop-lox YFP allele; All the HDC-cells are YFP-positive. Scale bars, 60 μm (top panel); 20 μm (bottom panel). (B) Left column: Nissl stains (purple) of the TMN area of littermate control and *HDC-ΔBmal1* mice; Middle and right columns: Immunocytochemical staining with GFP antibodies to TMN sections from *HDC-Cre* x *Rosa26-lox-stop-lox-YFP* mice; after this mouse cross, the YFP stain is selective for HDC-positive neurons; Scale bars, 40 μm. (C) Whole cell current-clamp recordings of VTM histaminergic neurons from littermate control and *HDC-ΔBmal1* mice in acute brain slices taken from animals in the sleep (day) phase and the active (night) phase. Measures of excitability (membrane potential, input conductance and the membrane time constant) were unaffected by either time of day or absence of BMAL1. DTM, diffuse tuberomammilary area; VTM, ventral tuberomammilary area. MTM, medial tuberomammilary area.

Figure S4.

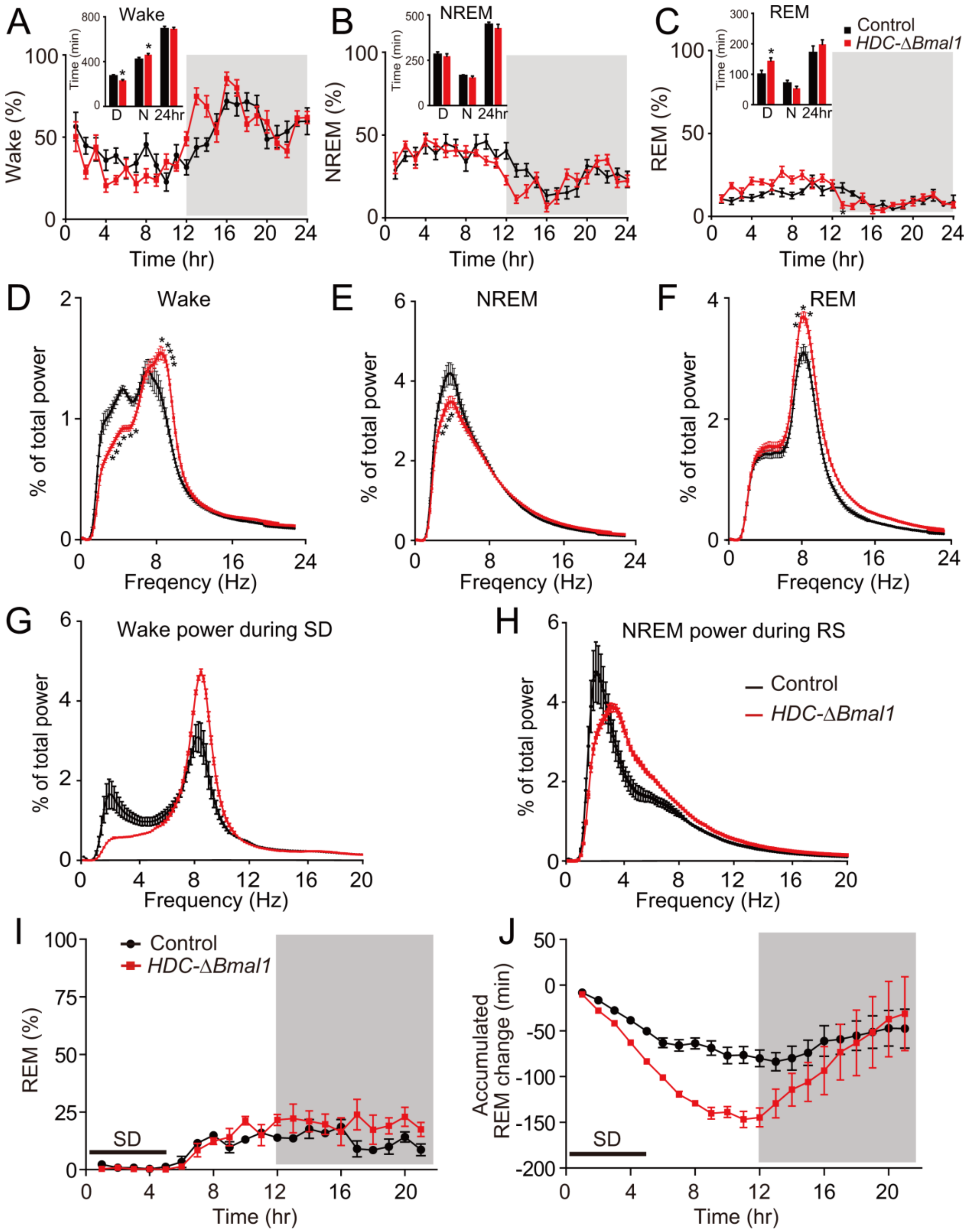

A
Wake
Wake (%)
Time (min)
D
N
24hr
Time (hr)
B
NREM
NREM (%)
C
REM
REM (%)
Control
HDC-ΔBmal1
D
Wake
% of total power
Freqency (Hz)
E
NREM
F
REM
G
Wake power during SD
Frequency (Hz)
H
NREM power during RS
I
SD
J
Accumulated REM change (min)

Figure S4. Additional sleep parameters in *HDC-ΔBmal1* and littermate control mice (related to Figures 3 and 4).

(A, D) over 24 hours, the total wake time of *HDC-ΔBmal1* mice did not differ from littermate control mice; however, *HDC-ΔBmal1* mice spent slightly less total time asleep in the day, and more time awake during the night (*$p < 0.05$); over the 24 hour period, the wake EEG of *HDC-ΔBmal1* mice was shifted significantly to theta frequencies compared with littermate controls (*$p < 0.05$), and the delta frequency ranges were reduced (*$p < 0.05$); (B, E) *HDC-ΔBmal1* mice did not differ from littermate controls in how much total NREM sleep they had in the day or in the night, although their average NREM power over 24 hours was reduced (*$p < 0.05$); (C, F) *HDC-ΔBmal1* mice had more REM sleep during the day (*$p < 0.05$), and the total power of REM was significantly higher over the 24-hour period (*$p < 0.05$). In all graphs, *HDC-ΔBmal1* data are shown in red; littermate control data are shown in black. D: day; N: night. (G) The total wake power during the 5-hour sleep deprivation. Control mice had two peaks at 2 Hz and 8 Hz; the *HDC-ΔBmal1* mice had more theta power peaking at 8 Hz than the control littermates. (H) The total NREM power during the recovery sleep: control littermates had 2 - 4 Hz NREM power, but the NREM power of *HDC-ΔBmal1* mice was shifted to higher frequencies with lower power. (I, J) Effect of sleep deprivation (SD) on amount (%) of REM sleep at each time point and the rate of re-accumulation of REM sleep per hour.

Supplemental Experimental Procedures

Ethics. All experiments were performed in accordance with the UK Home Office Animal Procedures Act (1986), and all procedures were approved by the Imperial College Ethical Review Committee.

Mouse genotyping. Experiments were performed on male mice homozygous for the conditional *Bmal1* allele (JAX mice stock 007668) and *HDC-Cre*-negative littermate controls. Some of the mice also contained a *Rosa26-lox-stop-lox-YFP* allele. Mice were genotyped by PCR analysis using ear DNA. The specific primers for genotyping were: iCre-forward: 5′-GTGTGGCTGCCCCTTCTGCC-3′; iCre-reverse: 5′-AGCCTCACCATGGCCCCAGT-3′ (250 bp product); Bmal1-forward: 5′-ACTGGAAGTAACTTTATCAAACTG-3′; Bmal1-reverse: 5′-CTGACCAACTTGCTAACAATTA-3′ (327 bp product wild-type; 431 bp product, floxed allele); RosaYFP-forward: 5′-GGC GCTACCGGTCGCCACCATGGTGAGCAAGGGCGAGGAGC-3′; RosaYFP-reverse: 5′-GCGCGCGTTAACTTACTTGTACAGCTCGTCCATGCC-3′ (980 bp product).

qPCR. Total RNA at the selected ZT points from the TMN, DLG or cerebellar area was extracted using Trizol (Invitrogen). TaqMan assay probes were designed by Life technologies (UK), and purchased from Invitrogen (UK). The probes were: mHDC Mm00456104_m1; mBmal1 Mm00500226_m1; mPer1 Mm00501813_m1; mCry1 Mm00514392_m1; mHnmt Mm00475563_m1; m18s rRNA Mm03928990_g1; mRev-erbα Mm00520708_m1.

Immunocytochemistry. Mice were transcardially perfused with 4% paraformaldehyde in PBS. Brains were removed and 35μm-thick coronal sections were cut using a Leica VT1000S vibratome. Free-floating sections were washed in PBS three times for 5 min, permeabilized in PBS plus 0.4% Triton X-100 for

30 min, blocked by incubation in PBS plus 5% normal goat serum (NGS), 0.2% Triton X-100 for 1 h (all at room temperature) and subsequently incubated with primary antibody which was diluted in PBS plus 2% NGS overnight or 48 h (HDC) at 4°C. Incubated slices were washed three times in PBS for 10 min at room temperature, incubated for 2 h at room temperature with a 1:1000 dilution of a secondary antibody (Molecular Probes) in PBS plus 1% NGS, and subsequently washed there times in PBS for 10 min at room temperature. Slices were mounted on slides, embedded in Mowiol (DAPI or without DAPI) (Vector, UK), cover-slipped, and analyzed using an upright fluorescent microscope (Nikon eclipse 80i, Nikon Corporation, Japan), a Zeiss LSM 510 confocal microscope or a Zeiss LSM 510 inverted confocal microscope (Facility for Imaging by Light Microscopy, Imperial College). Confocal images were acquired using single track scanning or Z-scan. Fluorescence intensity was quantified using ImageJ (NIH, USA).

Primary antisera used were rabbit polyclonal EGFP (Invitrogen Molecular Probes, UK), 1:1000; guinea pig polyclonal HDC (American Research Products, Belmont, MA), 1:300; rabbit polyclonal BMAL1, 1:1000; rabbit polyclonal PER2, 1:1000, (the BMAL1 and PER2 antisera were generated by the M. Hastings lab – antisera specificity has been confirmed on sections from BMAL1 and PER2 knockout brains); Secondary antibodies were Alexa Fluor 488 goat anti-rabbit IgG, 1:1000, Alexa Fluor 488 goat anti-guinea pig IgG, 1:1000, Alexa Fluor 594 goat anti-rabbit IgG, 1:1000, Alexa Fluor 594 goat anti-guinea pig IgG, 1:1000, (Invitrogen Molecular Probes, UK).

Histamine measurements. Brains were collected at ZT4 and ZT8 during the day and ZT16 and ZT20 during the night and homogenized with 10 μL of 0.2 M

perchloric acid per mg tissue and centrifuged at 10000 rpm for 5 minutes at 4°C. The supernatants were collected and neutralized with an equal volume of 1M potassium borate buffer. Brain histamine levels were determined with an ELISA kit (Beckman Coulter Co. # IM2562).

Luciferase assays. A proximal fragment of the mouse *hdc* gene upstream region was amplified by PCR from genomic DNA using the primers (forward: 5′-ATATATGAATTCCTTGGATTGCTCCTCCTGGCAGACAG-3′; reverse: 5′-ATATATGGTACCGACCACCCCCCATGAAGTCTGTTGTG-3′). To clone the *hdc* gene promoter into the pGL3-basic luciferase vector (Promega), primers (forward: 5′-CTACGTAGCCATGCTCTAGGTACCGACCAC- 3′; reverse: 5′-GACAAGCTTCGACGCGTGCTCTGAATTCCTTG-3′) were used to amplify a fragment from the mouse *hdc* gene promoter. The E-box in the *hdc* proximal promoter region was mutated in the *hdc-promoter-luciferase* vector by Quick Change mutagenesis (Agilent Technologies, UK). *Hdc* promoter (500 ng) or mutant promoter luciferase vectors (500 ng) and control pRL-sv40 Renilla luciferase (5 ng) (Promega, Cat.#E2231) were transiently transfected into the HEK293 cells using the calcium phosphate method, along with combinations of expression vectors for BMAL1 (50 ng or 100 ng), CLOCK (50 ng or 100 ng), CRY1 (100 ng or 200 ng) and PER2 (100 ng or 200 ng). PcDNA3.1 plasmids were added to each group to make the same amount of DNA before transfection. Luciferase activity was measured by the Dual-luciferase reporter assay system (Promega, UK), using a Lumat3 (LB 9508) instrument (Berthold Technologies).

Electrophysiology of acute slices. Adult male mice were killed by cervical dislocation. The brain was rapidly removed and immersed in ice-cold slicing solution (slicing ACSF contained the following in mM: 2.5 KCl, 1 $CaCl_2$, 5 MgCl,

1.25 NaH_2PO_4, 26 $NaHCO_3$ and 11 glucose) bubbled with 95%O_2/5%CO_2. The tissue block was cut between the cerebellum and optic tract, and brain slices were produced with a vibratome tissue slicer (Campden Instruments). Coronal slices were cut at a thickness of 250 μm and transferred to a holding chamber containing slicing ACSF continuously bubbled with 95%O_2/5%CO_2. Once slicing was complete, the holding chamber was transferred to a 37°C heat block for 15 min, after which the slicing ACSF was gradually exchanged for recording ACSF (which contained the following in mM: 125 NaCl, 2.5 KCl, 2 $CaCl_2$, 2 MgCl, 1.25 NaH_2PO_4, 26 $NaHCO_3$, and 11 glucose, pH 7.4 when bubbled with 95%O_2/5%CO_2) over 40 min, then allowed to reach room temperature before electrophysiological recording experiments.

A *ROSA26-lox-stop-lox-YFP* allele [1] was also present in the *HDC-ΔBmal1* mice. Neurons were visualized with primary GFP fluorescence using a fixed-stage upright microscope (Slice Scope Pro 1000, Scientifica) fitted with a high numerical aperture water-immersion objective (Olympus, 40×/0.8W LUMPlanFL N), and a digital camera. The recording chamber was continuously perfused with external solution via a gravity perfusion system at a rate of about 3 ml min^{-1}. Patch pipette resistances were typically <5 MΩ when back-filled with internal solution. We recorded in current-clamp using internal solutions containing the following (in mM): 145 K-gluconate, 4 NaCl, 5 KCl, 0.5 $CaCl_2$, 5 EGTA, 10 HEPES, 4 Mg-ATP, and 5 sucrose; the pH was adjusted to 7.3 with KOH. We used a current injection protocol (800-ms pulse, −40 to 60 pA in 2 pA increments) to confirm the presence of I_h and to examine the f-I (action potential frequency to current injection) relationship. The membrane time constant τ_m was calculated from a series of brief step pulses: 0.5-ms pulses, 100

pA to threshold. Cell capacitance and recording parameters were calculated from a 10-mV step in voltage clamp, from a holding potential of -60mV.

Locomotion, sleep-wake behavior, EEG and EMG electrode implantation. For the locomotion experiments, mice were put into an open box and allowed to free run. All experiments were performed after lights off for 6-8 hr when mice were in their waking phase. The locomotion activity was detected by infrared beams around the box. To habituate mice to the weight of EEG recording devices, sham Neurologgers were attached two days before the EEG recording session. All surgery was carried out under halothane (1.5–2.5% in oxygen) anesthesia. Three EEG electrodes (Decolletage, AG) were inserted through the skull onto the dura mater, the coordinates for the three electrodes were: frontal bone (+1.5 mm to Bregma, -1.5 mm from midline), parietal bone (+1.5 mm to Bregma, +1.5 mm from midline), interparietal bone (+2.0 mm from Lambda, 0.0 mm from midline). For EMG recording, three lengths of Teflon-insulated stainless steel wire were inserted in the neck muscle. After the three electrodes and EMG wires were in place, they were covered with dental cement (Orthoresin; DeguDent GmbH). The animals were allowed at least 14 days to recover from surgery.

Sleep deprivation. Mice were sleep deprived for 5 hours, starting at the beginning of “lights off”. At the first hour of the sleep deprivation, mice were put into novel cages with new objects. After each hour, objects were exchanged with new objects. During the final two hours, mice were gently handled for a few seconds if they appeared to sleep. Most of the time, the mice were active. In a total of two hours, the mice only needed to be handled only once or twice. After sleep deprivation, the mice were put back in their home cages.

EEG recording. The EEG and EMG signals were recorded using Neurologger 2 devices which enable non-tethered recording. Two days before the sleep experiments, mice were attached with sham Neurologgers. Sleep experiments were performed 2 to 3 hours before "lights off" and 1-2 hours after "lights off" the following day, a total of 27 to 29 hours data were recorded. Two EEG and two EMG channels were recorded. To analyze the EEG data, Spike2 (v7.10) (Cambridge Electronic Design) was used. The sampling rate was set up to 200 Hz. The EMG was filtered by band pass between 5 and 45 Hz. EEG frequency below 0.5 Hz was filtered by low pass. The sleep state (wake, non-rapid-eye-movement, rapid-eye-movement) was scored automatically as before (S8, 9), and then confirmed manually. EEG power was analyzed using FFT power spectra and Morlet Wavelet analysis.

Circadian analysis. For circadian recording, mice were housed in individual cages equipped with running-wheels with food and water available *ad libitum* in light-tight ventilated chambers. Adult male mice were maintained for 7–10 d in 12 h white light/ 12 h dim red light (LD) conditions and were subsequently transferred to continuous dim red light (DD) conditions for 10–14 d. Wheel running data were collected and analyzed using Clocklab (Actimetrics Software).

Novel Object Recognition. An open box (45 x 45 x 35 cm) was used to conduct the experiment. A camera was mounted above the open field. Before the experiment, mice were handled every day for one week. Two days before the experiment, they were allowed to explore the open box for 10 minutes to get familiar with the apparatus. Each test contained three sessions. In the first session, mice were placed into the empty open field without objects for 10 minutes. In the second session, two identical objects were added. Mice were

then placed into the open field for another 10 minutes to explore the objects. In the third session, the object presented was a duplicate of the sample presented in the second session to avoid olfactory trails, and a different object was introduced in the third session. In a control experiment (normal 24-hour natural sleep), the interval between second and third session was 24 hours. In the sleep deprivation (SD) experiment, after the second session, the mice were sleep deprived for five hours and allowed to recover for 19 hours in their home cages and after recovery sleep, the third session was performed.

[1] Srinivas, S., Watanabe, T., Lin, C.S., William, C.M., Tanabe, Y., Jessell, T.M., and Costantini, F. (2001) Cre reporter strains produced by targeted insertion of EYFP and ECFP in to the ROSA26 locus. BMC Dev Biol *1*, 4
