# Supplementary material for: Circadian Factor BMAL1 in Histaminergic Neurons Regulates Sleep Architecture
Source: Curr Biol. 2014 Dec 1;24(23):2838–44. doi: 10.1016/j.cub.2014.10.019 (PMC4252164; doi:10.1016/j.cub.2014.10.019)
Supplement: Document S2. Article plus Supplemental Information [file mmc2.pdf]

Current Biology 24, 2838–2844, December 1, 2014 ©2014 The Authors http://dx.doi.org/10.1016/j.cub.2014.10.019

Report

# Circadian Factor BMAL1 in Histaminergic Neurons Regulates Sleep Architecture

**Xiao Yu,[1] Anna Zecharia,[1] Zhe Zhang,[1] Qianzi Yang,[1] Raquel Yustos,[1] Polona Jager,[1] Alexei L. Vyssotski,[2] Elizabeth S. Maywood,[3] Johanna E. Chesham,[3] Ying Ma,[1] Stephen G. Brickley,[1] Michael H. Hastings,[3] Nicholas P. Franks,[1,4,*] and William Wisden[1,4,*]**
[1]Department of Life Sciences, Imperial College London, Sir Ernst Chain Building, Exhibition Road, London SW7 2AZ, UK
[2]Institute of Neuroinformatics, University of Zurich and ETH Zurich, Winterhurerstrasse 190, Zurich 8057, Switzerland
[3]Neurobiology Division, Medical Research Council Laboratory of Molecular Biology, Cambridge Biomedical Campus, Francis Crick Avenue, Cambridge CB2 0QH, UK

## Summary

**Circadian clocks allow anticipation of daily environmental changes [1]. The suprachiasmatic nucleus (SCN) houses the master clock, but clocks are also widely expressed elsewhere in the body [1]. Although some peripheral clocks have established roles [1], it is unclear what local brain clocks do [2, 3]. We tested the contribution of one putative local clock in mouse histaminergic neurons in the tuberomamillary nucleus to the regulation of the sleep-wake cycle. Histaminergic neurons are silent during sleep, and start firing after wake onset [4–6]; the released histamine, made by the enzyme histidine decarboxylase (HDC), enhances wakefulness [7–11]. We found that *hdc* gene expression varies with time of day. Selectively deleting the *Bmal1* (also known as *Arntl* or *Mop3* [12]) clock gene from histaminergic cells removes this variation, producing higher HDC expression and brain histamine levels during the day. The consequences include more fragmented sleep, prolonged wake at night, shallower sleep depth (lower nonrapid eye movement [NREM] δ power), increased NREM-to-REM transitions, hindered recovery sleep after sleep deprivation, and impaired memory. Removing BMAL1 from histaminergic neurons does not, however, affect circadian rhythms. We propose that for mammals with polyphasic/nonwake consolidating sleep, the local BMAL1-dependent clock directs appropriately timed declines and increases in histamine biosynthesis to produce an appropriate balance of wake and sleep within the overall daily cycle of rest and activity specified by the SCN.**

## Results and Discussion

### A Putative BMAL1-Driven Clock in Histaminergic Neurons

Tuberomamillary nucleus (TMN) neurons expressing the *histidine decarboxylase* (*hdc*) gene are the sole neuronal source of histamine [13–15]. The *hdc* gene shows haploinsufficiency: a 2-fold decrease in *hdc* mRNA levels halves the brain content of histamine in mice [16, 17], and in humans, having only one functional *hdc* allele produces a type of Tourette syndrome [16]. Thus, modest changes in *hdc* transcript levels in TMN neurons can change the amount of histamine released and influence behavior. Changes in *hdc* mRNA levels also seem to occur in the normal daily cycle. *hdc* mRNA levels in human hypothalamus are 1.6-fold higher for daytime deaths [18], and HDC enzyme activity and histamine levels in rat brain vary with time of day [19–21]. In agreement with these data, immunocytochemical staining for HDC protein in mouse TMN neurons was stronger at zeitgeber time (ZT)18 (night, mid-lights off, the period when the animals are more active) than at ZT6 (day, mid-lights on) (3.5 ± 0.19 versus 1 ± 0.09 arbitrary units [AUs]; unpaired two-tailed t test, $p < 0.001$) (Figures 1A and 1B). In control mice there was also a 1.5-fold variation in *hdc* transcript levels over 24 hr (unpaired two-tailed, t test, $p < 0.05$): *hdc* mRNA was highest at the start of the night (ZT12), declined during the night, and increased during the day (Figure 1C). By contrast, transcripts encoding the enzyme that inactivates histamine, histamine N-methyltransferase (HNMT), did not show daily variation in the TMN area (Figure 1C). This daily variation in HDC expression could indicate a clock-like mechanism in histaminergic neurons. Indeed, histaminergic neurons express the core clock protein BMAL1 (Figure 1D). (BMAL1 antisera specificity was confirmed by the absence of staining in suprachiasmatic nucleus [SCN] sections from *BMAL1* global knockout brains [Figure S1A available online].)

### Removing BMAL1 from Histaminergic Neurons Changes the Local Expression of Core Circadian Genes and Elevates *hdc* Expression

We crossed *HDC-Cre* mice [22] with animals containing a floxed *Bmal1* gene [23] (Figure S1B). The resulting *HDC-ΔBmal1* mice were similar to littermate controls in weight (control weight, 26.6 ± 0.6 g, n = 5; *HDC-ΔBmal1* weight, 27 ± 0.7 g, n = 5; unpaired two-tailed t test, $p = 0.34$) and seemed healthy. All the HDC-positive cells lost BMAL1 (Figure 1D). In our characterization of the *HDC-Cre* mice, we found that transient developmental expression of the *hdc* gene produced recombination in several additional places, in particular the dorsal lateral geniculate (DLG) thalamic nucleus, the ventral medial (VM) hypothalamic nucleus, and Purkinje neurons [22]. By immunostaining, there was no indication of BMAL1 loss from the DLG, VMH, and cerebellum of *HDC-ΔBmal1* brains (Figures S1C–S1E); *Bmal1* and *per1* transcript levels in these regions were also unchanged (two-way ANOVA and post hoc Bonferroni, $p > 0.05$) (Figure S1F). The failure to delete the *bmal1* gene in these areas likely reflects that the particular floxed allele is relatively Cre insensitive, requiring sustained doses of Cre to produce recombination [24].

BMAL1 could serve housekeeping functions unrelated to its clock role. To see whether removing BMAL1 from histaminergic neurons disrupted the local clock, we examined the expression of core clockwork-associated genes in the TMN of control and *HDC-ΔBmal1* mice. In littermate control mice, *Per1*, *Cry1*, and *Rev-erbα* mRNA levels peaked around the beginning of the night (Figure S1G); in *HDC-ΔBmal1* mice, the expression rhythms of these three genes across the light-dark cycle were flattened; *Per1* and *Cry1* mRNA levels were, on average, higher, whereas *Rev-erbα* levels were significantly lower

[4]Co-senior author
*Correspondence: n.franks@imperial.ac.uk (N.P.F.), w.wisden@imperial.ac.uk (W.W.)
This is an open access article under the CC BY license (http://creativecommons.org/licenses/by/3.0/).

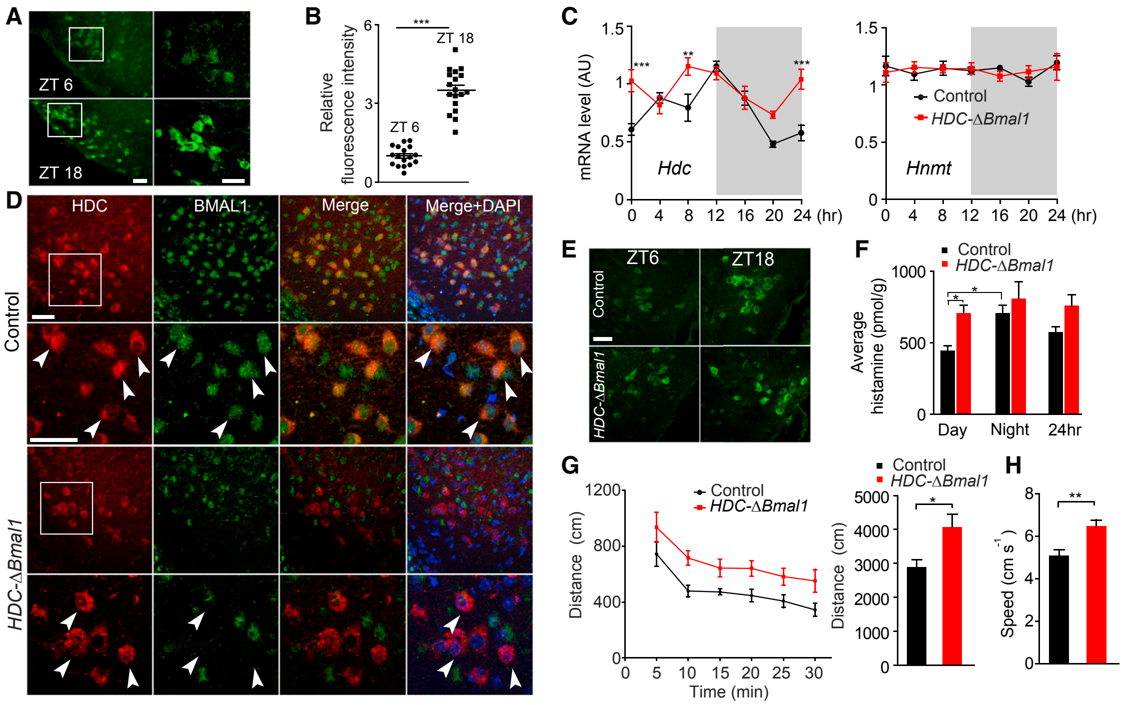


Figure 1. Rhythmic Expression of HDC and Histamine Requires BMAL1 in HDC-Positive Cells

(A and B) Expression of HDC in the mouse TMN varies with time of day. The intensity of immunocytochemical staining of neurons with antiserum to HDC (green) was higher at ZT18 than at ZT6 (n = 3 mice in both groups; n = 17 cells in control mice; n = 18 cells in *HDC-ΔBmal1* mice). Boxed regions in (A) are shown at higher power. The scale bars represent 40 μm. The graph (B) was obtained by imaging the fluorescence intensity of individual neurons; bars indicate SEM; ***p < 0.001.

(C) Quantitative PCR analysis of RNA from posterior hypothalamic tissue shows that transcripts encoding HDC vary with time of day, but in *HDC-ΔBmal1* mice (red traces) these rhythms were altered. HNMT transcript levels were unchanged; all transcript levels were normalized to expression of the *18S rRNA* gene. Bars represent SEM; **p < 0.01, ***p < 0.001.

(D) Histaminergic neurons in the TMN area, identified by immunocytochemistry with HDC (red), also contained BMAL1 protein (green); in *HDC-ΔBmal1* mice, BMAL1 staining was selectively removed. Magnifications are shown in the boxed regions. 4′,6-diamidino-2-phenylindole (DAPI) (blue) shows the position of cell nuclei. Arrowheads indicate examples of HDC-positive cells with BMAL1 (control) or without BMAL1 (*HDC-ΔBmal1* knockouts). The scale bars represent 40 μm.

(E) Compared with littermate controls, HDC protein is elevated at all ZT points in the TMN of *HDC-ΔBmal1* mice. The scale bar represents 40 μm.

(F) Average histamine levels are elevated in *HDC-ΔBmal1* brains during the day (bars represent SEM; *p < 0.05).

(G and H) *HDC-ΔBmal1* mice (red trace) traveled farther than littermate control mice in a 30 min period (G), and speed in total 30 min was higher (H) (n = 10 control; n = 10 *HDC-ΔBmal1*; bars represent SEM; *p < 0.05, **p < 0.01).

See also Figures S1–S3.

(Figure S1G) (two-way ANOVA and post hoc Bonferroni, *p < 0.05, **p < 0.01; Cosinor analysis [cosinor.exe, version 2.3; http://www.circadian.org/softwar.html]; *Per1*: control: amplitude, 0.63, p < 0.05; *HDC-ΔBmal1*: p = 0.27; *Cry1*: control: amplitude, 0.25, p < 0.05; *HDC-ΔBmal1*: p = 0.25; *Rev-erbα*: control: amplitude, 0.9, p = 0.01; *HDC-ΔBmal1*: amplitude, 0.29, p = 0.05; Cosinor p values are related to comparisons of goodness of cosine fit). Furthermore, the rhythmic expression of PER2 protein was abolished in histaminergic neurons in *HDC-ΔBmal1* mice (Figure S1H; the specificity of the PER2 antiserum was confirmed in *per2* knockout mice [25]). These results indicate that BMAL1 deletion from histaminergic neurons has likely disrupted their local clock mechanism.

In the *HDC-ΔBmal1* mice, *hdc* gene expression showed a disrupted 24 hr pattern (two-way ANOVA and post hoc Bonferroni, **p < 0.01, ***p < 0.001), and *hdc* transcript levels and protein were overall higher in the day and the late night. This produced increased brain histamine levels in the day (Figure 1F; two-way ANOVA or one-way ANOVA and post hoc Bonferroni, *p < 0.05). To test the behavioral consequence of upregulated *hdc* gene expression in TMN neurons, we examined locomotor activity in an open field. *HDC-ΔBmal1* mice traveled farther and at higher speeds (Figures 1G and 1H) than littermate controls (unpaired two-tailed t test, *p < 0.05, **p < 0.01).

BMAL1-CLOCK dimers can either activate or repress target genes [26, 27]. Is the *hdc* gene directly repressed by BMAL1? The 5′ region of the mouse *hdc* gene contains an E box. BMAL1-CLOCK dose-dependently increased *hdc promoter-luciferase* gene expression (Figure S2A) (one-way ANOVA and post hoc Bonferroni, ***p < 0.001), but not when the E box was mutated (Figure S2B). This was the opposite of the in vivo situation, when *hdc* transcript levels increased after BMAL1 deletion. Thus, in histaminergic neurons, BMAL1 could recruit a repressor complex onto the *hdc* promoter [27]. Alternatively, *RORE* sequences in the *hdc* gene could bind the repressor and core clock protein REV-ERBα [28, 29]. Diminished REV-ERBα levels in the TMN of *HDC-ΔBmal1* mice (Figure S1G) might derepress the *hdc* gene.

## Intrinsic Electrical Properties of Histaminergic Neurons Are Not Influenced by BMAL1

SCN neurons show cell-intrinsic circadian regulation of their electrophysiological parameters, partly determining when

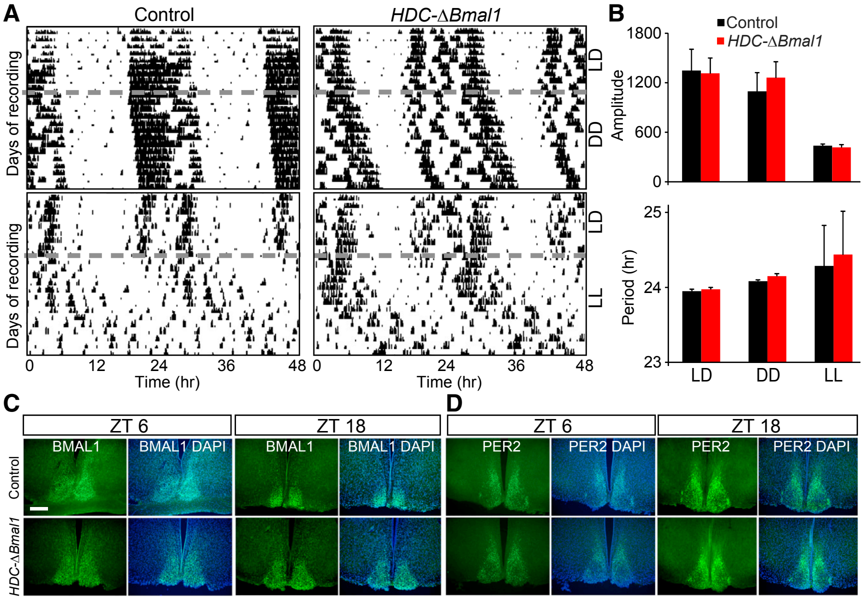


Figure 2. *HDC-ΔBmal1* Mice Have a Functionally Normal Circadian Clock

(A) Representative wheel-running actograms from homozygous *loxBmal1* mice and *HDC-ΔBmal1* mice. Mice were initially entrained to 12 h white light, 12 h dim red light (LD) and then transferred to continuous dim red light (DD) or continuous white light (LL).

(B) *HDC-ΔBmal1* mice (n = 6) and littermate controls (n = 8) did not have differing circadian periods or amplitudes during LD, LL, or DD (bars represent SEM; $p > 0.05$).

(C and D) Immunocytochemical analysis shows that the circadian rhythm of BMAL1 (green) and PER2 (green) expression is unaffected in the SCN of *HDC-ΔBmal1* mice. Sections are counterstained to show all cell nuclei with DAPI. The scale bar represents 0.5 mm.

these neurons fire [30–32]. We made whole-cell current-clamp recordings of histaminergic neurons from littermate and *HDC-ΔBmal1* mice during night and day (Figure S3C). Resting membrane potential, input conductance, current injection to threshold of action potential firing, capacitance, and membrane time constant were unaffected by time of day or the absence of BMAL1 (Figure S3C). We expect that *HDC-ΔBmal1* histaminergic neurons will fire action potentials normally but release more histamine.

### *HDC-ΔBmal1* Mice Have an Unchanged Circadian Wheel-Running Behavior

*HDC-ΔBmal1* knockout mice had an unchanged behavioral circadian rhythm and phase, compared with littermate controls, as assessed by wheel running in free-running conditions of constant darkness (DD) (unpaired two-tailed t test, $p > 0.05$) (Figures 2A and 2B) [25]. In free-running constant light (LL), both genotypes were more variable in period length than in LD or DD (Figure 2A). However, the amplitude of the peak period was lower and more variable in LL than in LD and DD, indicating the mice were equally less active in LL than in LD or DD, regardless of genotype (Figures 2A and 2B). Within the SCN, the circadian variation in BMAL1 and PER2 proteins was unchanged between *HDC-ΔBmal1* knockout mice and littermate controls (Figures 2C and 2D); there was little variation in BMAL1 staining intensity in the SCN between ZT6 and ZT18 (Figure 2C), highlighting that although BMAL1 is the core component of the clock, its levels change little during the circadian cycle. CLOCK and BMAL1 are often constitutively bound to E boxes. The critical rhythm for BMAL1-CLOCK activity arises from PER-CRY, which arrives to inhibit, and then dissociates from, the BMAL1-CLOCK complex [33]. PER2 staining in the SCN of both groups of mice increased at ZT18 compared with ZT6 (Figure 2D). Thus, the *HDC-ΔBmal* mice had an unaffected SCN molecular clock and circadian pace making.

### *HDC-ΔBmal1* Mice Have More Fragmented Sleep

Mice unable to synthesize histamine (*HDC* knockouts) show normal sleep-wake behavior throughout most of the 24 hr cycle, except they are significantly less awake just before, and for the first few hours after, the start of the night [10]. It is intriguing that *HDC* knockout mice have a selective deficit in *anticipating* lights off, further suggesting a circadian involvement of histaminergic neurons. In contrast to *HDC* knockout mice, the *HDC-ΔBmal1* mice have a gain of function in the histaminergic system. We looked at the consequences for the sleep-wake cycle (Figure 3; Figure S4). Sleep experiments and nontethered electroencephalogram (EEG) analysis were performed using Neurologger2 devices [22, 34]. For the first part of the night, *HDC-ΔBmal1* mice were more awake, as assessed by electromyogram (EMG) and the ratio of $\delta:\theta$ power in the EEG, than littermate controls (Figures S4A–S4C), namely the opposite of *HDC* knockout mice [10]. As the night progressed, the EEG:EMG ratios of the *HDC-ΔBmal1* mice became similar to littermate controls (Figure S4A). Some the *HDC-ΔBmal1* mice had long (up to 40 min) periods of uninterrupted waking (Figure 3B). The total wake time, however, of *HDC-ΔBmal1* mice averaged over 24 hr was unchanged (693 ± 21 min versus 693 ± 12 min, unpaired two-tailed t test, $p > 0.05$), but over the night they spent more time awake than littermate control mice and less time awake during the day (night: 420 ± 16 min versus 461 ± 10 min, unpaired two-tailed t test, $p < 0.05$; day: 273 ± 9 min versus 231 ± 7 min, unpaired two-tailed t test, $p < 0.05$) (Figure S4A). Throughout the 24 hr, during the wake periods, the *HDC-ΔBmal1* mice had higher $\theta$ frequencies in the EEG than littermate controls (two-way ANOVA and post hoc Bonferroni, $p < 0.05$) (Figure S4D).

The amount of nonrapid eye movement (NREM) sleep was similar between *HDC-ΔBmal1* and control mice (Figure S4B) (488 ± 11 min versus 427 ± 20 min, unpaired two-tailed t test, $p > 0.05$), but NREM power was lower (Figure S4E; see next section) (two-way ANOVA and post hoc Bonferroni, *$p < 0.05$). During the day, *HDC-ΔBmal1* mice had more NREM episodes than controls (Figure 3C), but these episodes were shorter (Figure 3D) (3.5 ± 0.3 min versus 2.4 ± 0.3 min, unpaired two-tailed t test, **$p < 0.01$). The amount of REM sleep in *HDC-ΔBmal1* mice compared with littermate controls was higher in the day (Figure S4C): there were more episodes (Figure 3E), although episode duration was unchanged (Figure 3F) (1.7 ± 0.04 min versus 1.8 ± 0.04 min, unpaired two-tailed t test, $p > 0.05$); however, REM episode duration was shorter in the *HDC-ΔBmal1*

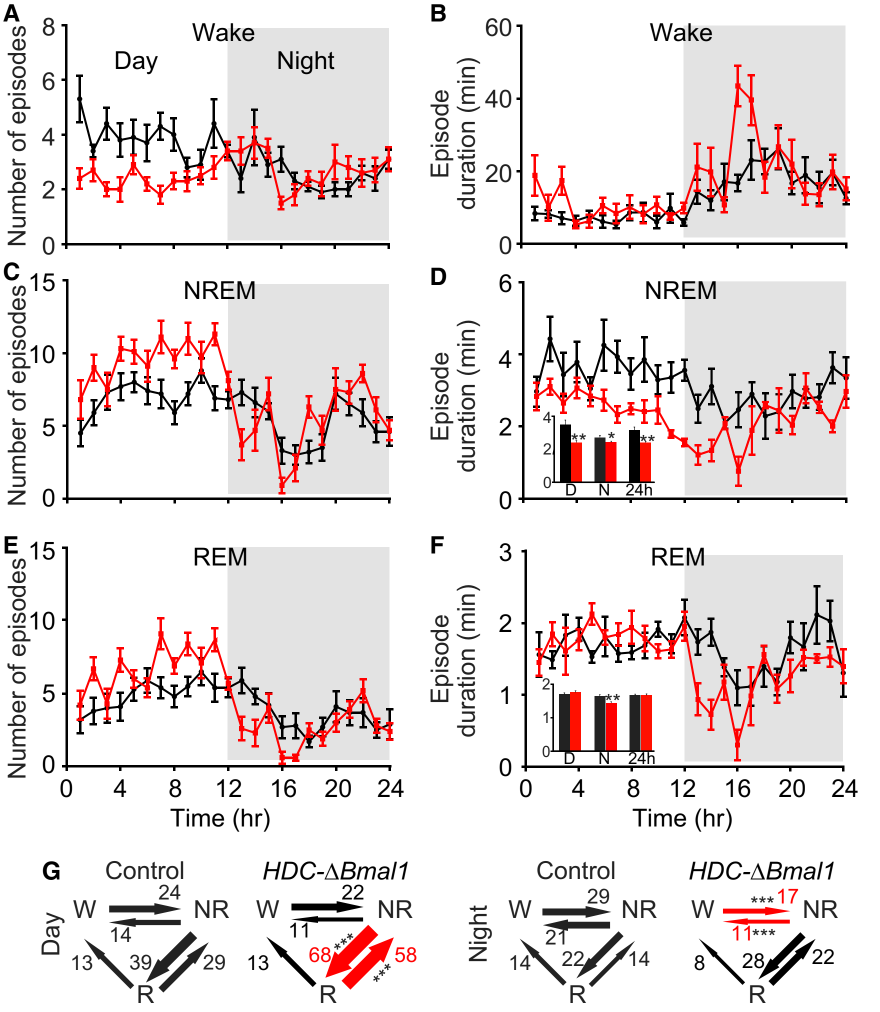


Figure 3. *HDC-⊿Bmal1* Mice Have Fragmented Sleep

(A–F) The number of vigilance (wake, NREM, and REM) episodes (A, C, and E, respectively) and episode duration (B, D, and F, respectively) over the 24 hr interval for *HDC-⊿Bmal1* mice (n = 10) (red traces) and littermate controls (n = 10). Bars represent SEM; *p < 0.05, **p < 0.01. D, day; N, night.

(G) Number of transitions between wake (W), NREM (NR), and REM (R) sleep in the day and night. Significant differences in transition numbers are shown with red arrows; ***p < 0.001. See also Figure S4.

mice (Figure 3F) during the night (1.6 ± 0.04 min versus 1.4 ± 0.03 min, unpaired two-tailed t test, **$p < 0.01$).

The daytime sleep architecture of *HDC-⊿Bmal1* mice differed from littermate control mice (Figure 3G). *HDC-⊿Bmal1* mice had more "NREM-to-REM" (39 ± 2 versus 68 ± 2, unpaired two-tailed t test, ***$p < 0.001$) and "REM-to-NREM" (29 ± 1 versus 58 ± 2, unpaired two-tailed t test, ***$p < 0.001$) transitions during the day (Figure 3G). During the night, there was no difference between genotypes in NREM-REM transitions (22 ± 3 versus 28 ± 2, unpaired two-tailed t test, $p > 0.05$) (Figure 3G); however, *HDC-⊿Bmal1* mice had fewer wake-to-NREM transitions (29 ± 3 versus 17 ± 1, unpaired two-tailed t test, ***$p < 0.001$) and vice versa (21 ± 3 versus 11 ± 1, unpaired two-tailed t test, ***$p < 0.001$) (Figure 3G), reflecting that they were awake more (Figure 3B). Thus, sustained elevated histamine in *HDC-⊿Bmal1* mice changed the sleep-wake architecture.

## After Sleep Deprivation, the Recovery Sleep of *HDC-⊿Bmal1* Mice Is Shorter with Less Power

*HDC-⊿Bmal1* mice and littermate controls were sleep deprived for 5 hr during the start of the day [35]. Mice were placed into a novel cage, and objects (plastic tubes, pieces of paper) were introduced that were exchanged each hour. This method reliably prevented sleep (Figure 4A). Consistent with the raised brain histamine levels in *HDC-⊿Bmal1* mice, the EEG profiles between the genotypes differed during sleep deprivation: littermate control mice had frequencies distributed in the δ-to-θ range (2–10 Hz), with two peaks at 2 and 8 Hz, but the *HDC-⊿Bmal1* mice had a single broad peak of enhanced power relative to controls, centered in the θ range (Figures S4G and S4H).

After sleep deprivation, mice slept freely in their home cages. Littermate control mice had a recovery sleep lasting about 10–12 hr (Figure 4A), with sustained NREM periods remaining 6 hr into the night. They reaccumulated their NREM sleep at a rate of approximately 30 min extra NREM sleep per hour (Figure 4B). The δ power in the EEG of littermate control mice remained elevated, compared with presleep deprivation levels, for some 12 hr after sleep deprivation (Figure 4C). By contrast, *HDC-⊿Bmal1* mice did not sustain their recovery sleep: it was about 6 hr shorter than sleep-deprived control littermates (Figure 4A), and their enhanced δ power of recovery sleep, already lower compared with littermate controls before sleep deprivation, remained lower as it declined to baseline (Figure 4C; Figure S4H). *HDC-⊿Bmal1* mice reaccumulated their NREM sleep at a slower rate than control mice: 12.5 min extra NREM sleep per hour (Figure 4B). Because *HDC-⊿Bmal1* mice had more REM baseline sleep than littermate controls during the day (Figure 3G), they had more REM loss during sleep deprivation, namely they had more REM sleep to lose by sleep deprivation (Figure S4J). *HDC-⊿Bmal1* mice also had a quicker reaccumulation of REM sleep during recovery sleep (Figure S4J). In the recovery stage after sleep deprivation, *HDC-⊿Bmal1* mice had more transitions from NREM to REM, which caused more REM gain but less NREM gain. The reason could be that *hdc* expression was stronger in the *HDC-⊿Bmal1* mice during recovery sleep (see next section).

## HDC Expression Is Reversibly Elevated by Sleep Deprivation and Requires BMAL1 to Reduce Its Expression to Baseline

We examined HDC expression in TMN neurons at the end of the sleep deprivation period (ZT5; 5 hr of sleep deprivation).

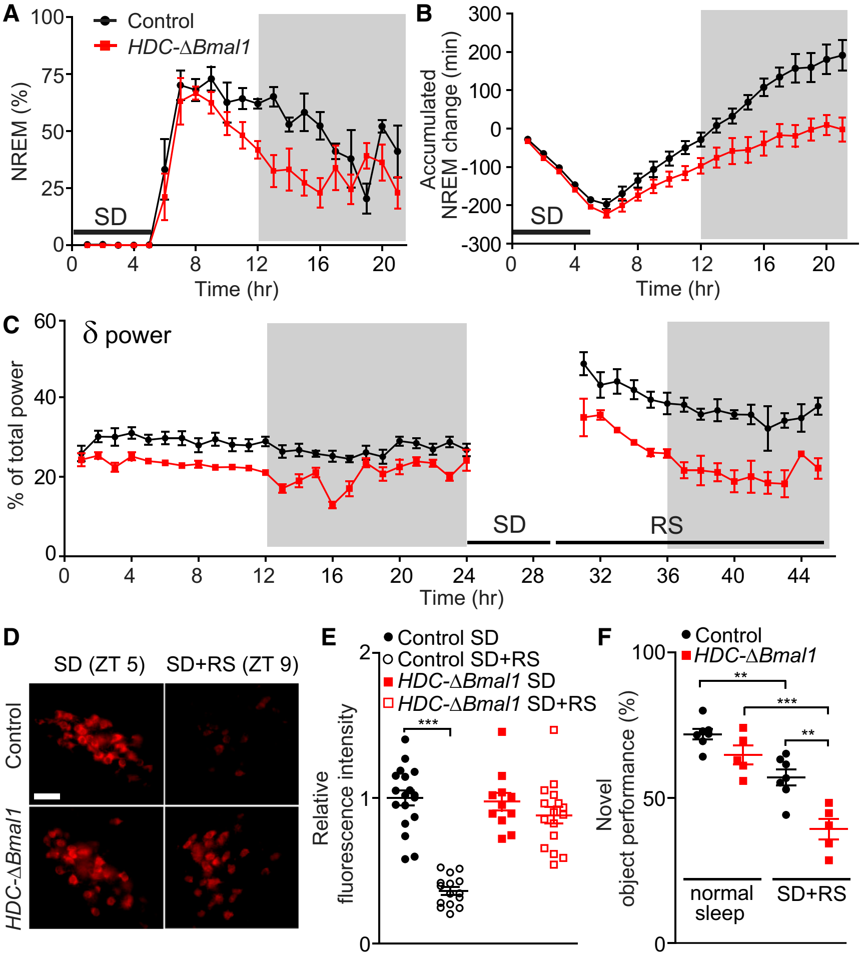


Figure 4. *HDC-ΔBmal1* Mice Have Compromised Recovery Sleep and Elevated HDC Expression after Sleep Deprivation and Compromised Novel Object Recognition

(A) After 5 hr of sleep deprivation (SD) during the day, littermate control mice (n = 5) had sustained NREM sleep; in contrast, the recovery sleep time of the *HDC-ΔBmal1* mice (red trace; n = 5) was reduced.

(B) Rate of accumulated NREM change following SD.

(C) Graph of NREM δ power of *HDC-ΔBmal1* mice and littermate controls before and after sleep deprivation. RS, recovery sleep.

(D) HDC immunocytochemical staining in littermate control and *HDC-ΔBmal1* mice at the end of SD (ZT5) and following 4 hr of recovery sleep (ZT9). The scale bar represents 40 μm.

(E) Quantification of relative HDC expression following sleep deprivation and recovery sleep. Each point represents an HDC-positive neuron. Bars represent SEM; ***p < 0.001.

(F) Control littermates (n = 7) and *HDC-ΔBmal1* mice (n = 5) were trained for 10 min to explore the same object, and then the mice were allowed 22 hr of normal sleep-wake behavior or subjected to 5 hr of sleep deprivation and allowed 17 hr of recovery sleep. One of the objects was then exchanged with a new object, and the mice were again tested for 10 min. Both control littermates and *HDC-ΔBmal1* mice performed worse after SD + RS, compared with those that had only a normal sleep-wake experience; however, *HDC-ΔBmal1* mice performed less well. The "novel object performance" was defined as the time exploring the novel object divided by the time exploring the familiar object and novel object, expressed as a percentage. Bars represent SEM; **p < 0.01, ***p < 0.001.

See also Figure S4.

ZT5 is when HDC and histamine levels are normally lower (Figure 1). At the end of the deprivation period, however, HDC expression was at the higher nighttime levels in both littermate controls and *HDC-ΔBmal1* mice (Figure 4D), suggesting that sleep deprivation increases *hdc* gene expression. Consistently, sleep deprivation raises histamine levels in cerebrospinal fluid [36]. In control mice, 4 hr into recovery sleep, HDC protein expression had decreased (roughly halved) to typical ZT6 levels (1% ± 0.05% versus 0.36% ± 0.03%, AUs, one-way ANOVA and post hoc Bonferroni, ***p < 0.001) (Figure 4E). In *HDC-ΔBmal1* mice, HDC protein expression remained elevated (Figure 4E). Presumably, in wild-type mice, *hdc* expression increases to combat the effects of sleep deprivation, and BMAL1 represses *hdc* gene expression back to baseline levels, ensuring good recovery sleep. Without BMAL1 in histaminergic neurons, the *hdc* gene expression level stayed flat and higher because it was already high before sleep deprivation and could not be further induced.

### *HDC-ΔBmal1* Mice Are More Impaired in Novel Object Recognition following Sleep Deprivation and Recovery Sleep

We investigated whether the diminished recovery sleep after sleep deprivation of *HDC-ΔBmal1* mice affected their ability at novel object recognition (Figure 4F). In mice, this memory task is sensitive to sleep deprivation [35, 37]. Control littermates and *HDC-ΔBmal1* mice were tested [35] either during the night phase of their normal sleep-wake cycle or after 5 hr of sleep deprivation followed by 17 hr of recovery sleep. Mice were trained for 10 min in the open field with the same objects; control littermates and *HDC-ΔBmal1* mice spent equal time exploring the two objects. In normal sleep-wake cycle conditions, control littermates and *HDC-ΔBmal1* mice performed the same (72% ± 2% versus 65% ± 3%, one-way ANOVA and post hoc Bonferroni, p > 0.05) (Figure 4F). For both genotypes, sleep deprivation impaired performance in recognizing the novel object, even after 17 hr of recovery sleep (Figure 4F); however, *HDC-ΔBmal1* mice performed worse (56% ± 3% versus 39% ± 3%, one-way ANOVA and post hoc Bonferroni, **p < 0.01) (Figure 4F). Thus, the reduced recovery of NREM sleep in *HDC-ΔBmal1* mice, compared to littermate controls, impaired cognitive function (Figures 4A and 4B).

## Conclusions

Circadian transcription factors regulate arousal and sleep [3, 12, 38, 39]. Our work reveals a specified function for local clock factors in histaminergic circuitry controlling arousal. BMAL1 in histaminergic neurons promotes a daily 1.5-fold fluctuation in *hdc* gene expression, with lower mRNA levels during the day. We propose that the local BMAL1-dependent clock mechanism suppresses daytime histaminergic tone and thereby facilitates appropriately timed intervals of sleep

and wake synchronized to the animal's overall circadian behavior.

### Supplemental Information

Supplemental Information includes Supplemental Experimental Procedures and four figures and can be found with this article online at http://dx.doi.org/10.1016/j.cub.2014.10.019.

### Acknowledgments

This work was funded by grants from the Wellcome Trust (S.G.B., N.P.F., and W.W.), Medical Research Council (G0800399, W.W.; G0901892, N.P.F., S.G.B., and W.W.; Laboratory of Molecular Biology core support, M.H.H.), Biotechnology and Biological Sciences Research Council (BB/K018159/1, W.W., S.G.B., M.H.H., and N.P.F.), and a UK-China Scholarships for Excellence/China Scholarship Council scheme (X.Y.). We thank Charles J. Weitz (Harvard Medical School) for depositing the floxed Bmal1 mouse line at the Jackson Laboratory. We also thank Wei Pan (Department of Bioengineering, Imperial College London) for helping with the sleep analysis.

Received: June 30, 2014
Revised: August 29, 2014
Accepted: October 8, 2014
Published: November 13, 2014

### References

1. Mohawk, J.A., Green, C.B., and Takahashi, J.S. (2012). Central and peripheral circadian clocks in mammals. Annu. Rev. Neurosci. *35*, 445–462.
2. Kyriacou, C.P., and Hastings, M.H. (2010). Circadian clocks: genes, sleep, and cognition. Trends Cogn. Sci. *14*, 259–267.
3. Franken, P. (2013). A role for clock genes in sleep homeostasis. Curr. Opin. Neurobiol. *23*, 864–872.
4. Takahashi, K., Lin, J.S., and Sakai, K. (2006). Neuronal activity of histaminergic tuberomammillary neurons during wake-sleep states in the mouse. J. Neurosci. *26*, 10292–10298.
5. Saper, C.B., Fuller, P.M., Pedersen, N.P., Lu, J., and Scammell, T.E. (2010). Sleep state switching. Neuron *68*, 1023–1042.
6. Lin, J.S., Anaclet, C., Sergeeva, O.A., and Haas, H.L. (2011). The waking brain: an update. Cell. Mol. Life Sci. *68*, 2499–2512.
7. Lin, J.S., Sakai, K., and Jouvet, M. (1988). Evidence for histaminergic arousal mechanisms in the hypothalamus of cat. Neuropharmacology *27*, 111–122.
8. Haas, H., and Panula, P. (2003). The role of histamine and the tuberomamillary nucleus in the nervous system. Nat. Rev. Neurosci. *4*, 121–130.
9. Anaclet, C., Parmentier, R., Ouk, K., Guidon, G., Buda, C., Sastre, J.P., Akaoka, H., Sergeeva, O.A., Yanagisawa, M., Ohtsu, H., et al. (2009). Orexin/hypocretin and histamine: distinct roles in the control of wakefulness demonstrated using knock-out mouse models. J. Neurosci. *29*, 14423–14438.
10. Parmentier, R., Ohtsu, H., Djebbara-Hannas, Z., Valatx, J.L., Watanabe, T., and Lin, J.S. (2002). Anatomical, physiological, and pharmacological characteristics of histidine decarboxylase knock-out mice: evidence for the role of brain histamine in behavioral and sleep-wake control. J. Neurosci. *22*, 7695–7711.
11. Zant, J.C., Rozov, S., Wigren, H.K., Panula, P., and Porkka-Heiskanen, T. (2012). Histamine release in the basal forebrain mediates cortical activation through cholinergic neurons. J. Neurosci. *32*, 13244–13254.
12. Bunger, M.K., Wilsbacher, L.D., Moran, S.M., Clendenin, C., Radcliffe, L.A., Hogenesch, J.B., Simon, M.C., Takahashi, J.S., and Bradfield, C.A. (2000). Mop3 is an essential component of the master circadian pacemaker in mammals. Cell *103*, 1009–1017.
13. Watanabe, T., Taguchi, Y., Hayashi, H., Tanaka, J., Shiosaka, S., Tohyama, M., Kubota, H., Terano, Y., and Wada, H. (1983). Evidence for the presence of a histaminergic neuron system in the rat brain: an immunohistochemical analysis. Neurosci. Lett. *39*, 249–254.
14. Panula, P., Yang, H.Y., and Costa, E. (1984). Histamine-containing neurons in the rat hypothalamus. Proc. Natl. Acad. Sci. USA *81*, 2572–2576.
15. Bayliss, D.A., Wang, Y.M., Zahnow, C.A., Joseph, D.R., and Millhorn, D.E. (1990). Localization of histidine decarboxylase mRNA in rat brain. Mol. Cell. Neurosci. *1*, 3–9.
16. Castellan Baldan, L., Williams, K.A., Gallezot, J.D., Pogorelov, V., Rapanelli, M., Crowley, M., Anderson, G.M., Loring, E., Gorczyca, R., Billingslea, E., et al. (2014). Histidine decarboxylase deficiency causes Tourette syndrome: parallel findings in humans and mice. Neuron *81*, 77–90.
17. Ohtsu, H., Tanaka, S., Terui, T., Hori, Y., Makabe-Kobayashi, Y., Pejler, G., Tchougounova, E., Hellman, L., Gertsenstein, M., Hirasawa, N., et al. (2001). Mice lacking histidine decarboxylase exhibit abnormal mast cells. FEBS Lett. *502*, 53–56.
18. Shan, L., Hofman, M.A., van Wamelen, D.J., Van Someren, E.J., Bao, A.M., and Swaab Dick, F. (2012). Diurnal fluctuation in histidine decarboxylase expression, the rate limiting enzyme for histamine production, and its disorder in neurodegenerative diseases. Sleep *35*, 713–715.
19. Orr, E., and Quay, W.B. (1975). Hypothalamic 24-hour rhythms in histamine, histidine, decarboxylase and histamine-N-methyltransferase. Endocrinology *96*, 941–945.
20. Rozov, S.V., Zant, J.C., Karlstedt, K., Porkka-Heiskanen, T., and Panula, P. (2014). Periodic properties of the histaminergic system of the mouse brain. Eur. J. Neurosci. *39*, 218–228.
21. Prast, H., Dietl, H., and Philippu, A. (1992). Pulsatile release of histamine in the hypothalamus of conscious rats. J. Auton. Nerv. Syst. *39*, 105–110.
22. Zecharia, A.Y., Yu, X., Götz, T., Ye, Z., Carr, D.R., Wulff, P., Bettler, B., Vyssotski, A.L., Brickley, S.G., Franks, N.P., and Wisden, W. (2012). GABAergic inhibition of histaminergic neurons regulates active waking but not the sleep-wake switch or propofol-induced loss of consciousness. J. Neurosci. *32*, 13062–13075.
23. Storch, K.F., Paz, C., Signorovitch, J., Raviola, E., Pawlyk, B., Li, T., and Weitz, C.J. (2007). Intrinsic circadian clock of the mammalian retina: importance for retinal processing of visual information. Cell *130*, 730–741.
24. Husse, J., Zhou, X., Shostak, A., Oster, H., and Eichele, G. (2011). Synaptotagmin10-Cre, a driver to disrupt clock genes in the SCN. J. Biol. Rhythms *26*, 379–389.
25. Meng, Q.J., Maywood, E.S., Bechtold, D.A., Lu, W.Q., Li, J., Gibbs, J.E., Dupré, S.M., Chesham, J.E., Rajamohan, F., Knafels, J., et al. (2010). Entrainment of disrupted circadian behavior through inhibition of casein kinase 1 (CK1) enzymes. Proc. Natl. Acad. Sci. USA *107*, 15240–15245.
26. Kondratov, R.V., Shamanna, R.K., Kondratova, A.A., Gorbacheva, V.Y., and Antoch, M.P. (2006). Dual role of the CLOCK/BMAL1 circadian complex in transcriptional regulation. FASEB J. *20*, 530–532.
27. Nguyen, K.D., Fentress, S.J., Qiu, Y., Yun, K., Cox, J.S., and Chawla, A. (2013). Circadian gene Bmal1 regulates diurnal oscillations of Ly6C(hi) inflammatory monocytes. Science *341*, 1483–1488.
28. Kojetin, D.J., and Burris, T.P. (2014). REV-ERB and ROR nuclear receptors as drug targets. Nat. Rev. Drug Discov. *13*, 197–216.
29. Preitner, N., Damiola, F., Lopez-Molina, L., Zakany, J., Duboule, D., Albrecht, U., and Schibler, U. (2002). The orphan nuclear receptor REV-ERB$\alpha$ controls circadian transcription within the positive limb of the mammalian circadian oscillator. Cell *110*, 251–260.
30. Belle, M.D., Diekman, C.O., Forger, D.B., and Piggins, H.D. (2009). Daily electrical silencing in the mammalian circadian clock. Science *326*, 281–284.
31. Colwell, C.S. (2011). Linking neural activity and molecular oscillations in the SCN. Nat. Rev. Neurosci. *12*, 553–569.
32. Granados-Fuentes, D., Norris, A.J., Carrasquillo, Y., Nerbonne, J.M., and Herzog, E.D. (2012). I(A) channels encoded by Kv1.4 and Kv4.2 regulate neuronal firing in the suprachiasmatic nucleus and circadian rhythms in locomotor activity. J. Neurosci. *32*, 10045–10052.
33. Forger, D.B., and Peskin, C.S. (2003). A detailed predictive model of the mammalian circadian clock. Proc. Natl. Acad. Sci. USA *100*, 14806–14811.
34. Vyssotski, A.L., Dell'Omo, G., Dell'Ariccia, G., Abramchuk, A.N., Serkov, A.N., Latanov, A.V., Loizzo, A., Wolfer, D.P., and Lipp, H.P. (2009). EEG responses to visual landmarks in flying pigeons. Curr. Biol. *19*, 1159–1166.
35. Halassa, M.M., Florian, C., Fellin, T., Munoz, J.R., Lee, S.Y., Abel, T., Haydon, P.G., and Frank, M.G. (2009). Astrocytic modulation of sleep homeostasis and cognitive consequences of sleep loss. Neuron *61*, 213–219.
36. Soya, A., Song, Y.H., Kodama, T., Honda, Y., Fujiki, N., and Nishino, S. (2008). CSF histamine levels in rats reflect the central histamine neurotransmission. Neurosci. Lett. *430*, 224–229.

37. Palchykova, S., Winsky-Sommerer, R., Meerlo, P., Dürr, R., and Tobler, I. (2006). Sleep deprivation impairs object recognition in mice. Neurobiol. Learn. Mem. *85*, 263–271.
38. Laposky, A., Easton, A., Dugovic, C., Walisser, J., Bradfield, C., and Turek, F. (2005). Deletion of the mammalian circadian clock gene BMAL1/Mop3 alters baseline sleep architecture and the response to sleep deprivation. Sleep *28*, 395–409.
39. Naylor, E., Bergmann, B.M., Krauski, K., Zee, P.C., Takahashi, J.S., Vitaterna, M.H., and Turek, F.W. (2000). The circadian clock mutation alters sleep homeostasis in the mouse. J. Neurosci. *20*, 8138–8143.

Current Biology, Volume *24*

Supplemental Information

# Circadian Factor BMAL1 in Histaminergic Neurons Regulates Sleep Architecture

Xiao Yu, Anna Zecharia, Zhe Zhang, Qianzi Yang, Raquel Yustos, Polona Jager, Alexei L. Vyssotski, Elizabeth S. Maywood, Johanna E. Chesham, Ying Ma, Stephen G. Brickley, Michael H. Hastings, Nicholas P. Franks, and William Wisden

## Supplemental Information

## Supplemental Figures and Legends

### Figure S1

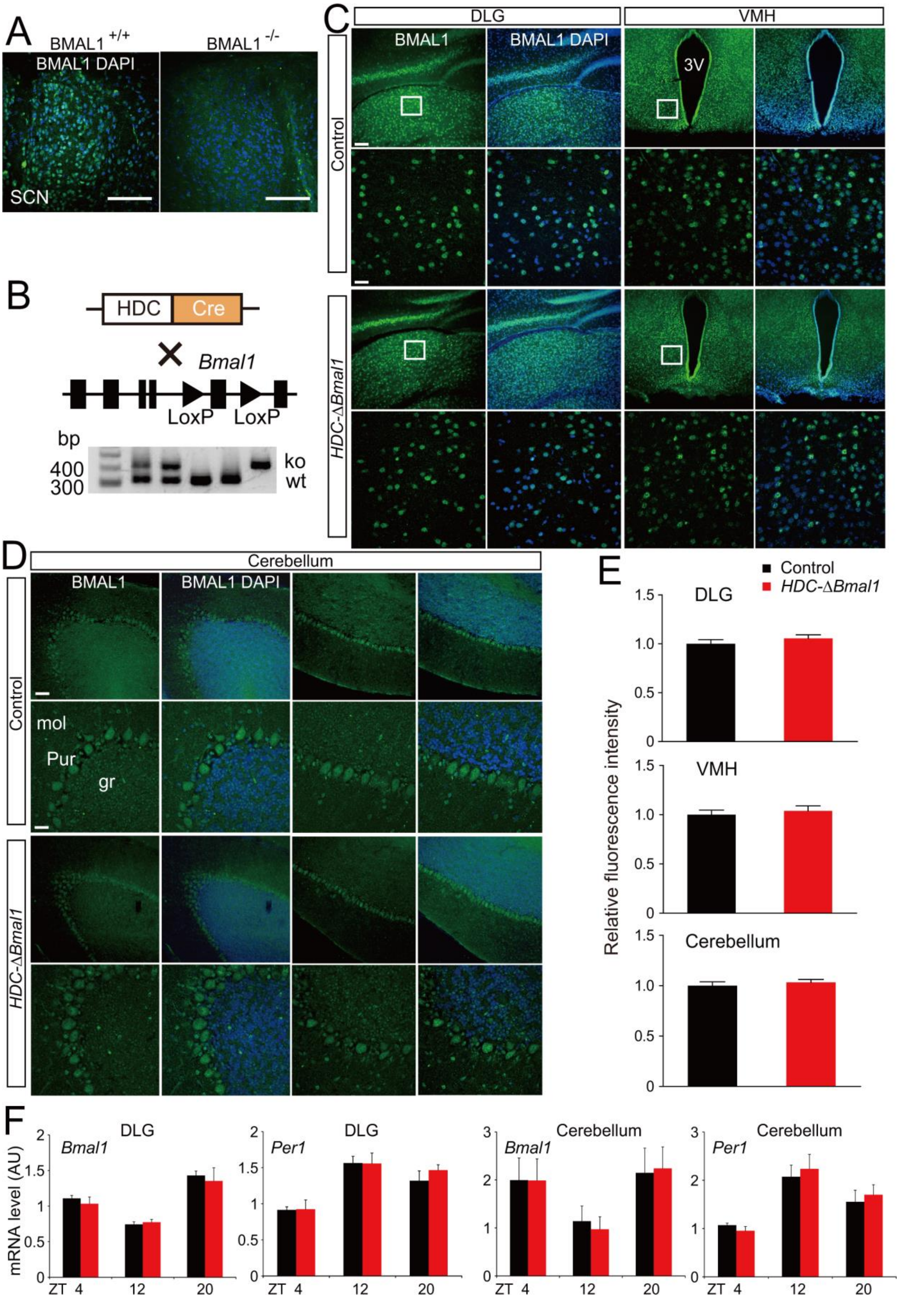

A
BMAL1 +/+
BMAL1 -/-
BMAL1 DAPI
SCN
B
HDC
Cre
Bmal1
LoxP
LoxP
bp
400
300
ko
wt
C
DLG
VMH
BMAL1
BMAL1 DAPI
3V
Control
HDC-ΔBmal1
D
Cerebellum
BMAL1
BMAL1 DAPI
Control
mol
Pur
gr
HDC-ΔBmal1
E
Control
HDC-ΔBmal1
DLG
VMH
Cerebellum
Relative fluorescence intensity
F
DLG
Bmal1
Per1
Cerebellum
mRNA level (AU)
ZT 4
12
20

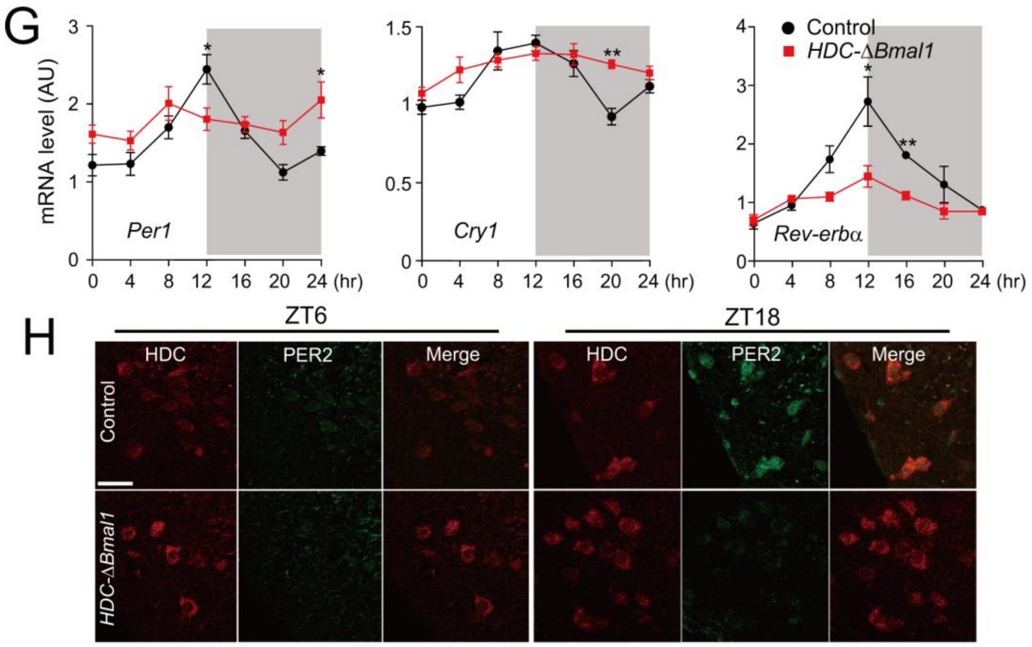


**Figure S1. Deletion of BMAL1 from histaminergic neurons disrupts a local clock (related to Figure 1)**

(A) To test the specificity of the BMAL1 antibody, SCN brain sections from BMAL1+/+ or BMAL1-/- mice (*loxBmal1* x *Nestin-Cre*) were incubated with the BMAL1 antisera. BMAL1 was detected in control mouse SCN cells but not in BMAL1-/- sections. Scale bar, 100 μm. (B) *HDC-Cre* mediated deletion of the 5$^{th}$ coding exon of the *Bmal1* gene; illustrative PCR genotyping using ear genomic DNA from crosses of *HDC-Cre/loxBmal1* x *loxBmal1/loxBmal1* mice. (C, D) BMAL1 protein expression as seen by immunocytochemistry was unaffected in the dorsal lateral geniculate thalamic (DLG) nucleus, the ventral medial hypothalamus (VMH) (panel C) and the cerebellar Purkinje cells and granule cells (panel D); Pur, Purkinje cells; gr, granule cells; mol, molecular layer. Green images are staining with florescent secondary antibodies to anti-Bmal1 antisera; the blue is a DAPI stain showing all cell nuclei. Scale bars, 0.5 mm (top panel); 20 μm (bottom panel). (E) Flourescence intensity of individual cells was

quantified in DLG ($n$ = 30 control vs. $n$ = 30 *HDC-ΔBmal1*), VMH ($n$ = 40 control vs. $n$ = 40 *HDC-ΔBmal1*) and cerebellum ($n$ = 21 control vs. $n$ = 20 *HDC-ΔBmal1*) using ImageJ; bars represent SEM, $p > 0.05$. (F) Q-PCR analysis of RNA from thalamus tissue (DLG punch) and cerebellum shows that transcripts encoding BMAL1 and PER1 did not show any difference in either genotype, $p > 0.05$). (G) Q-PCR analysis of RNA from posterior hypothalamic tissue (TMN punch) shows that transcripts encoding PER1, CRY1 and REV-ERB$\alpha$ vary with time of day; in *HDC-ΔBmal1* mice (red traces), these rhythms were altered. All transcript levels were normalized to expression of the *18s rRNA* gene. AU, arbitrary units; bars represent SEM, *$p < 0.05$, **$p < 0.01$. (H) In control HDC-positive neurons (staining with HDC antisera shown in red), PER2 immunoreactivity was low at ZT6 and was high at ZT18 (quite similar to the situation in the SCN, see Fig. 2). In HDC neurons in *HDC-ΔBmal1* mice, PER2 staining did not increase at ZT18. Scale bar, 40 μm.

**Figure S2**.

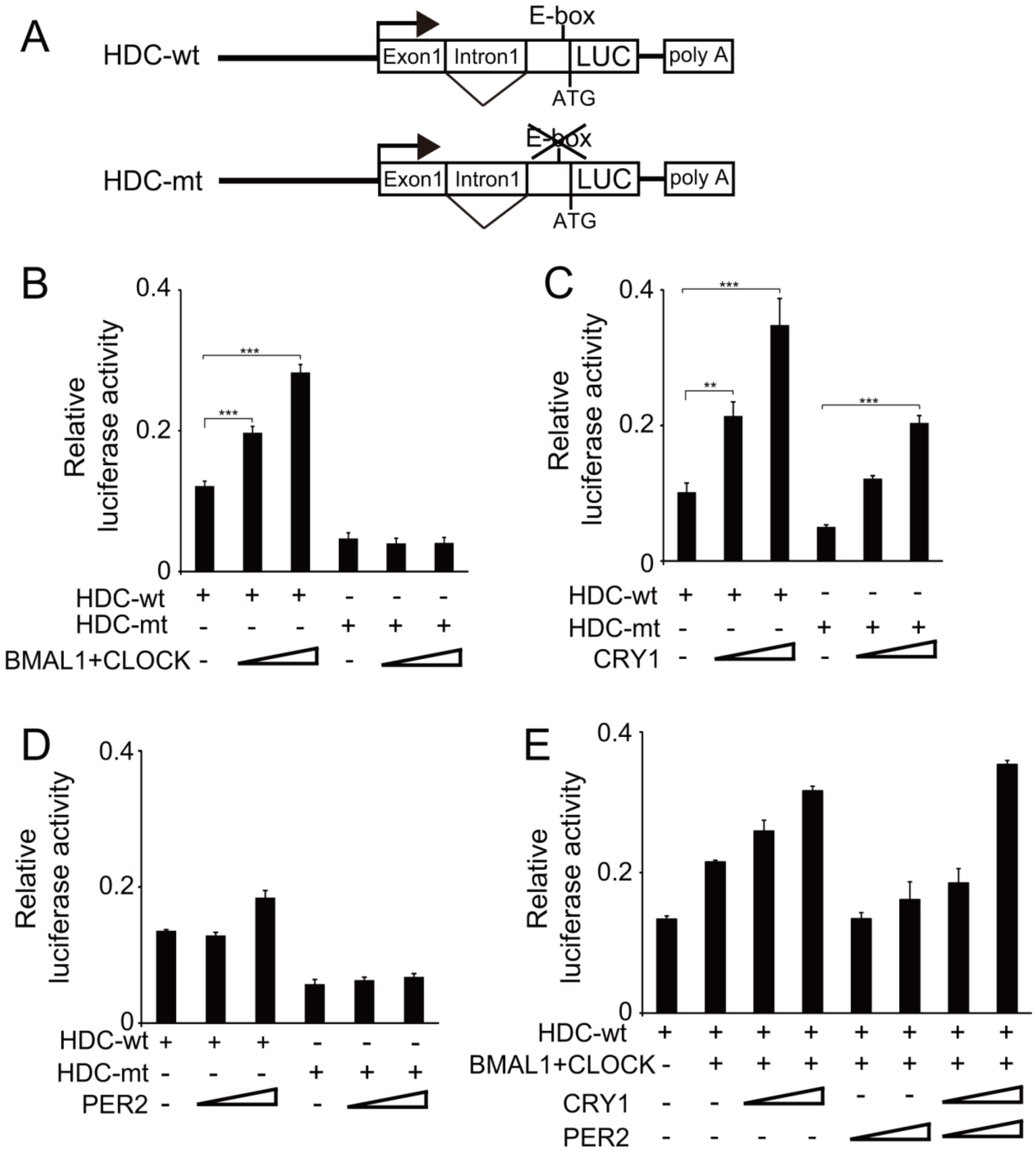


**Figure S2**. **The mouse *hdc* gene has a canonical E-box in the proximal promoter region which can be activated by BMAL:CLOCK dimers (related to Figure 1).** (A) Transient transfection assays in HEK cells were performed with two luciferase (LUC) reporter gene constructs, containing either the *hdc*

gene proximal promoter with the wild-type (HDC-wt) or mutated E-box (HDC-mt), together with co-transfection of specific combinations of expression plasmids for BMAL1, CLOCK, PER2 and CRY1; (B) The HDC-wt construct had a certain basal activity in HEK cells, even without the addition of exogenous BMAL1:CLOCK; however, this basal expression depended on the E-box, because mutation of the E-box abolished basal expression. Thus presumably the HDC-wt construct is activated to some extent by endogenous BMAL:CLOCK complexes in HEK cells. However, exogenous BMAL1:CLOCK co-transfection increased HDC-wt expression in direct proportion to the amount of BMAL:CLOCK added; in contrast, HDC-mt expression was not increased by BMAL:CLOCK co-transfection, demonstrating the requirement of the E-box (***$p < 0.001$). (C) Because, *cry1* gene expression also increases in *HDC-ΔBmal1* mice (Figure S1G), as found in other BMAL1 deletion studies, we tested if CRY1 protein could activate the *hdc* promoter fragment. CRY1 dose-dependently stimulated *hdc promoter-luciferase* expression, even when the *E*-box was mutated (**$p < 0.01$, ***$p < 0.001$). Thus it could be that the elevated *cry1* gene expression in *HDC-ΔBmal1* neurons drives increased *hdc* gene transcription; (D) the *hdc* proximal promoter activity was unaffected by PER2. (E) CRY1 can still activate the *hdc* promoter even when PER2 is over expressed. Bars represent SEM.

**Figure S3.**

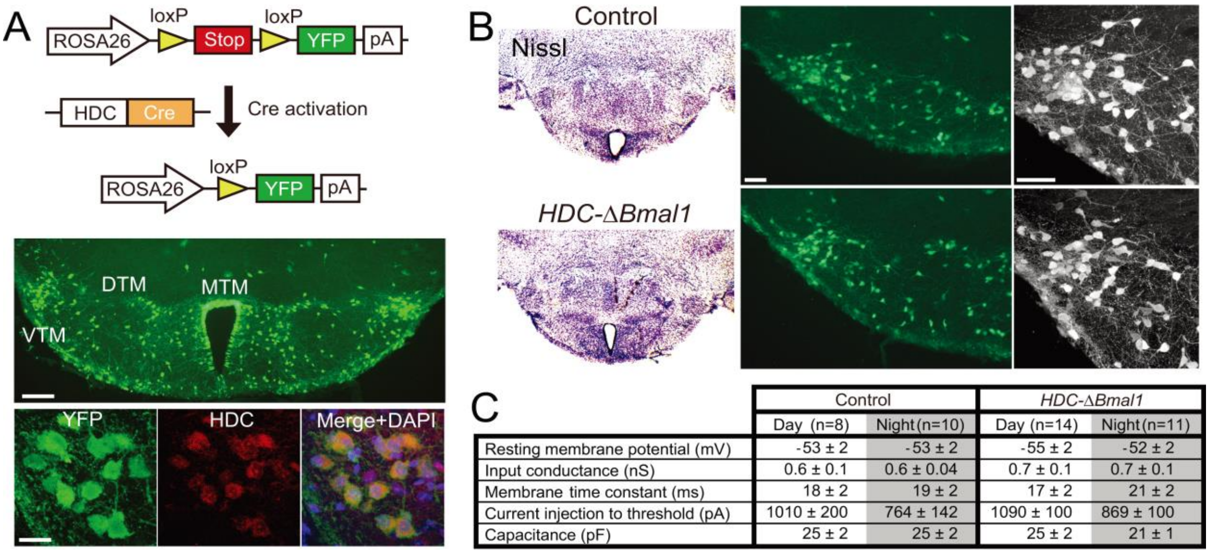


| | Control | | HDC-ΔBmal1 | |
|---|---|---|---|---|
| | Day (n=8) | Night(n=10) | Day (n=14) | Night(n=11) |
| Resting membrane potential (mV) | -53 ± 2 | -53 ± 2 | -55 ± 2 | -52 ± 2 |
| Input conductance (nS) | 0.6 ± 0.1 | 0.6 ± 0.04 | 0.7 ± 0.1 | 0.7 ± 0.1 |
| Membrane time constant (ms) | 18 ± 2 | 19 ± 2 | 17 ± 2 | 21 ± 2 |
| Current injection to threshold (pA) | 1010 ± 200 | 764 ± 142 | 1090 ± 100 | 869 ± 100 |
| Capacitance (pF) | 25 ± 2 | 25 ± 2 | 25 ± 2 | 21 ± 1 |

**Figure S3. Intrinsic electrical properties of histaminergic neurons are not influenced by BMAL1 (related to Figure 1).** (A) Histaminergic neurons in the TMN were visualized by crossing *HDC-Cre* mice with mice containing a Rosa26-lox-stop-lox YFP allele; All the HDC-cells are YFP-positive. Scale bars, 60 μm (top panel); 20 μm (bottom panel). (B) Left column: Nissl stains (purple) of the TMN area of littermate control and *HDC-ΔBmal1* mice; Middle and right columns: Immunocytochemical staining with GFP antibodies to TMN sections from *HDC-Cre* x *Rosa26-lox-stop-lox-YFP* mice; after this mouse cross, the YFP stain is selective for HDC-positive neurons; Scale bars, 40 μm. (C) Whole cell current-clamp recordings of VTM histaminergic neurons from littermate control and *HDC-ΔBmal1* mice in acute brain slices taken from animals in the sleep (day) phase and the active (night) phase. Measures of excitability (membrane potential, input conductance and the membrane time constant) were unaffected by either time of day or absence of BMAL1. DTM, diffuse tuberomammilary area; VTM, ventral tuberomammilary area. MTM, medial tuberomammilary area.

**Figure S4.**

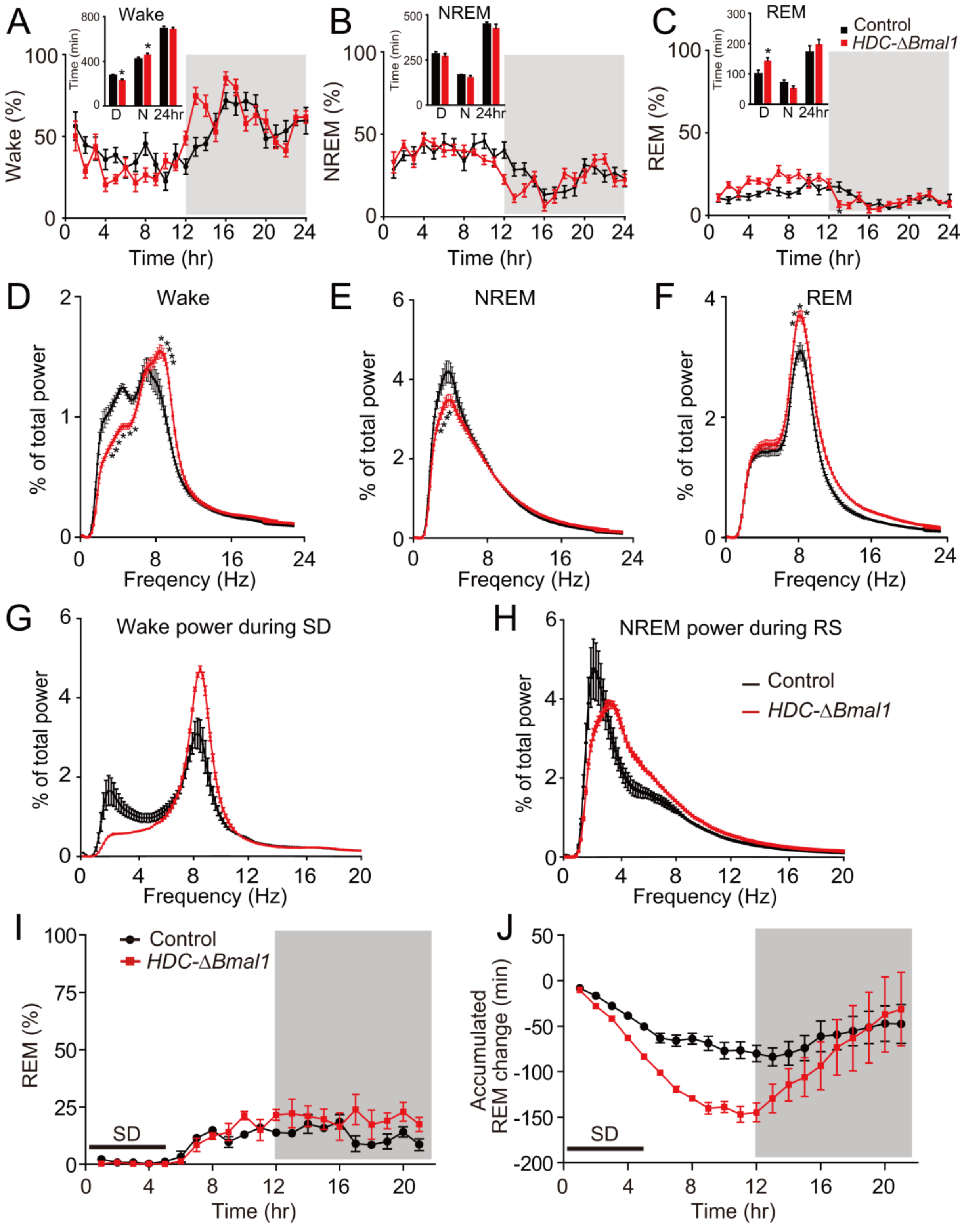
A
Wake
Wake (%)
Time (min)
D N 24hr
Time (hr)
B
NREM
NREM (%)
C
REM
REM (%)
Control
HDC-ΔBmal1
D
Wake
% of total power
Freqency (Hz)
E
NREM
F
REM
G
Wake power during SD
Frequency (Hz)
H
NREM power during RS
I
REM (%)
SD
J
Accumulated REM change (min)

**Figure S4. Additional sleep parameters in *HDC-ΔBmal1* and littermate control mice (related to Figures 3 and 4).**

(A, D) over 24 hours, the total wake time of *HDC-ΔBmal1* mice did not differ from littermate control mice; however, *HDC-ΔBmal1* mice spent slightly less total time asleep in the day, and more time awake during the night (*$p < 0.05$); over the 24 hour period, the wake EEG of *HDC-ΔBmal1* mice was shifted significantly to theta frequencies compared with littermate controls (*$p < 0.05$), and the delta frequency ranges were reduced (*$p < 0.05$); (B, E) *HDC-ΔBmal1* mice did not differ from littermate controls in how much total NREM sleep they had in the day or in the night, although their average NREM power over 24 hours was reduced (*$p < 0.05$); (C, F) *HDC-ΔBmal1* mice had more REM sleep during the day (*$p < 0.05$), and the total power of REM was significantly higher over the 24-hour period (*$p < 0.05$). In all graphs, *HDC-ΔBmal1* data are shown in red; littermate control data are shown in black. D: day; N: night. (G) The total wake power during the 5-hour sleep deprivation. Control mice had two peaks at 2 Hz and 8 Hz; the *HDC-ΔBmal1* mice had more theta power peaking at 8 Hz than the control littermates. (H) The total NREM power during the recovery sleep: control littermates had 2 - 4 Hz NREM power, but the NREM power of *HDC-ΔBmal1* mice was shifted to higher frequencies with lower power. (I, J) Effect of sleep deprivation (SD) on amount (%) of REM sleep at each time point and the rate of re-accumulation of REM sleep per hour.

**Supplemental Experimental Procedures**

**Ethics.** All experiments were performed in accordance with the UK Home Office Animal Procedures Act (1986), and all procedures were approved by the Imperial College Ethical Review Committee.

**Mouse genotyping.** Experiments were performed on male mice homozygous for the conditional *Bmal1* allele (JAX mice stock 007668) and *HDC-Cre*-negative littermate controls. Some of the mice also contained a *Rosa26-lox-stop-lox-YFP* allele. Mice were genotyped by PCR analysis using ear DNA. The specific primers for genotyping were: iCre-forward: 5′-GTGTGGCTGCCCCTTCTGCC-3′; iCre-reverse: 5′-AGCCTCACCATGGCCCCAGT-3′ (250 bp product); Bmal1-forward: 5′-ACTGGAAGTAACTTTATCAAACTG-3′; Bmal1-reverse: 5′-CTGACCAACTTGCTAACAATTA-3′ (327 bp product wild-type; 431 bp product, floxed allele); RosaYFP-forward: 5′-GGC GCTACCGGTCGCCACCATGGTGAGCAAGGGCGAGGAGC-3′; RosaYFP-reverse: 5′-GCGCGCGTTAACTTACTTGTACAGCTCGTCCATGCC-3′ (980 bp product).

**qPCR**. Total RNA at the selected ZT points from the TMN, DLG or cerebellar area was extracted using Trizol (Invitrogen). TaqMan assay probes were designed by Life technologies (UK), and purchased from Invitrogen (UK). The probes were: mHDC Mm00456104_m1; mBmal1 Mm00500226_m1; mPer1 Mm00501813_m1; mCry1 Mm00514392_m1; mHnmt Mm00475563_m1; m18s rRNA Mm03928990_g1; mRev-erbα Mm00520708_m1.

**Immunocytochemistry.** Mice were transcardially perfused with 4% paraformaldehyde in PBS. Brains were removed and 35μm-thick coronal sections were cut using a Leica VT1000S vibratome. Free-floating sections were washed in PBS three times for 5 min, permeabilized in PBS plus 0.4% Triton X-100 for

30 min, blocked by incubation in PBS plus 5% normal goat serum (NGS), 0.2% Triton X-100 for 1 h (all at room temperature) and subsequently incubated with primary antibody which was diluted in PBS plus 2% NGS overnight or 48 h (HDC) at 4°C. Incubated slices were washed three times in PBS for 10 min at room temperature, incubated for 2 h at room temperature with a 1:1000 dilution of a secondary antibody (Molecular Probes) in PBS plus 1% NGS, and subsequently washed there times in PBS for 10 min at room temperature. Slices were mounted on slides, embedded in Mowiol (DAPI or without DAPI) (Vector, UK), cover-slipped, and analyzed using an upright fluorescent microscope (Nikon eclipse 80i, Nikon Corporation, Japan), a Zeiss LSM 510 confocal microscope or a Zeiss LSM 510 inverted confocal microscope (Facility for Imaging by Light Microscopy, Imperial College). Confocal images were acquired using single track scanning or Z-scan. Fluorescence intensity was quantified using ImageJ (NIH, USA).

Primary antisera used were rabbit polyclonal EGFP (Invitrogen Molecular Probes, UK), 1:1000; guinea pig polyclonal HDC (American Research Products, Belmont, MA), 1:300; rabbit polyclonal BMAL1, 1:1000; rabbit polyclonal PER2, 1:1000, (the BMAL1 and PER2 antisera were generated by the M. Hastings lab – antisera specificity has been confirmed on sections from BMAL1 and PER2 knockout brains); Secondary antibodies were Alexa Fluor 488 goat anti-rabbit IgG, 1:1000, Alexa Fluor 488 goat anti-guinea pig IgG, 1:1000, Alexa Fluor 594 goat anti-rabbit IgG, 1:1000, Alexa Fluor 594 goat anti-guinea pig IgG, 1:1000, (Invitrogen Molecular Probes, UK).

**Histamine measurements.** Brains were collected at ZT4 and ZT8 during the day and ZT16 and ZT20 during the night and homogenized with 10 μL of 0.2 M

perchloric acid per mg tissue and centrifuged at 10000 rpm for 5 minutes at 4°C. The supernatants were collected and neutralized with an equal volume of 1M potassium borate buffer. Brain histamine levels were determined with an ELISA kit (Beckman Coulter Co. # IM2562).

**Luciferase assays.** A proximal fragment of the mouse *hdc* gene upstream region was amplified by PCR from genomic DNA using the primers (forward: 5′-ATATATGAATTCCTTGGATTGCTCCTCCTGGCAGACAG-3′; reverse: 5′-ATATATGGTACCGACCACCCCCCATGAAGTCTGTTGTG-3′). To clone the *hdc* gene promoter into the pGL3-basic luciferase vector (Promega), primers (forward: 5′-CTACGTAGCCATGCTCTAGGTACCGACCAC- 3′; reverse: 5′-GACAAGCTTCGACGCGTGCTCTGAATTCCTTG-3′) were used to amplify a fragment from the mouse *hdc* gene promoter. The E-box in the *hdc* proximal promoter region was mutated in the *hdc-promoter-luciferase* vector by Quick Change mutagenesis (Agilent Technologies, UK). *Hdc* promoter (500 ng) or mutant promoter luciferase vectors (500 ng) and control pRL-sv40 Renilla luciferase (5 ng) (Promega, Cat.#E2231) were transiently transfected into the HEK293 cells using the calcium phosphate method, along with combinations of expression vectors for BMAL1 (50 ng or 100 ng), CLOCK (50 ng or 100 ng), CRY1 (100 ng or 200 ng) and PER2 (100 ng or 200 ng). PcDNA3.1 plasmids were added to each group to make the same amount of DNA before transfection. Luciferase activity was measured by the Dual-luciferase reporter assay system (Promega, UK), using a Lumat3 (LB 9508) instrument (Berthold Technologies).

**Electrophysiology of acute slices.** Adult male mice were killed by cervical dislocation. The brain was rapidly removed and immersed in ice-cold slicing solution (slicing ACSF contained the following in mM: 2.5 KCl, 1 $CaCl_2$, 5 MgCl,

1.25 $NaH_2PO_4$, 26 $NaHCO_3$ and 11 glucose) bubbled with 95%$O_2$/5%$CO_2$. The tissue block was cut between the cerebellum and optic tract, and brain slices were produced with a vibratome tissue slicer (Campden Instruments). Coronal slices were cut at a thickness of 250 μm and transferred to a holding chamber containing slicing ACSF continuously bubbled with 95%$O_2$/5%$CO_2$. Once slicing was complete, the holding chamber was transferred to a 37°C heat block for 15 min, after which the slicing ACSF was gradually exchanged for recording ACSF (which contained the following in mM: 125 NaCl, 2.5 KCl, 2 $CaCl_2$, 2 MgCl, 1.25 $NaH_2PO_4$, 26 $NaHCO_3$, and 11 glucose, pH 7.4 when bubbled with 95%$O_2$/5%$CO_2$) over 40 min, then allowed to reach room temperature before electrophysiological recording experiments.

A *ROSA26-lox-stop-lox-YFP* allele [1] was also present in the *HDC-ΔBmal1* mice. Neurons were visualized with primary GFP fluorescence using a fixed-stage upright microscope (Slice Scope Pro 1000, Scientifica) fitted with a high numerical aperture water-immersion objective (Olympus, 40×/0.8W LUMPlanFL N), and a digital camera. The recording chamber was continuously perfused with external solution via a gravity perfusion system at a rate of about 3 ml $min^{-1}$. Patch pipette resistances were typically <5 MΩ when back-filled with internal solution. We recorded in current-clamp using internal solutions containing the following (in mM): 145 K-gluconate, 4 NaCl, 5 KCl, 0.5 $CaCl_2$, 5 EGTA, 10 HEPES, 4 Mg-ATP, and 5 sucrose; the pH was adjusted to 7.3 with KOH. We used a current injection protocol (800-ms pulse, −40 to 60 pA in 2 pA increments) to confirm the presence of $I_h$ and to examine the f-I (action potential frequency to current injection) relationship. The membrane time constant $\tau_m$ was calculated from a series of brief step pulses: 0.5-ms pulses, 100

pA to threshold. Cell capacitance and recording parameters were calculated from a 10-mV step in voltage clamp, from a holding potential of -60mV.

**Locomotion, sleep-wake behavior, EEG and EMG electrode implantation.** For the locomotion experiments, mice were put into an open box and allowed to free run. All experiments were performed after lights off for 6-8 hr when mice were in their waking phase. The locomotion activity was detected by infrared beams around the box. To habituate mice to the weight of EEG recording devices, sham Neurologgers were attached two days before the EEG recording session. All surgery was carried out under halothane (1.5–2.5% in oxygen) anesthesia. Three EEG electrodes (Decolletage, AG) were inserted through the skull onto the dura mater, the coordinates for the three electrodes were: frontal bone (+1.5 mm to Bregma, -1.5 mm from midline), parietal bone (+1.5 mm to Bregma, +1.5 mm from midline), interparietal bone (+2.0 mm from Lambda, 0.0 mm from midline). For EMG recording, three lengths of Teflon-insulated stainless steel wire were inserted in the neck muscle. After the three electrodes and EMG wires were in place, they were covered with dental cement (Orthoresin; DeguDent GmbH). The animals were allowed at least 14 days to recover from surgery.

**Sleep deprivation**. Mice were sleep deprived for 5 hours, starting at the beginning of “lights off”. At the first hour of the sleep deprivation, mice were put into novel cages with new objects. After each hour, objects were exchanged with new objects. During the final two hours, mice were gently handled for a few seconds if they appeared to sleep. Most of the time, the mice were active. In a total of two hours, the mice only needed to be handled only once or twice. After sleep deprivation, the mice were put back in their home cages.

**EEG recording.** The EEG and EMG signals were recorded using Neurologger 2 devices which enable non-tethered recording. Two days before the sleep experiments, mice were attached with sham Neurologgers. Sleep experiments were performed 2 to 3 hours before "lights off" and 1-2 hours after "lights off" the following day, a total of 27 to 29 hours data were recorded. Two EEG and two EMG channels were recorded. To analyze the EEG data, Spike2 (v7.10) (Cambridge Electronic Design) was used. The sampling rate was set up to 200 Hz. The EMG was filtered by band pass between 5 and 45 Hz. EEG frequency below 0.5 Hz was filtered by low pass. The sleep state (wake, non-rapid-eye-movement, rapid-eye-movement) was scored automatically as before (S8, 9), and then confirmed manually. EEG power was analyzed using FFT power spectra and Morlet Wavelet analysis.

**Circadian analysis.** For circadian recording, mice were housed in individual cages equipped with running-wheels with food and water available *ad libitum* in light-tight ventilated chambers. Adult male mice were maintained for 7–10 d in 12 h white light/ 12 h dim red light (LD) conditions and were subsequently transferred to continuous dim red light (DD) conditions for 10–14 d. Wheel running data were collected and analyzed using Clocklab (Actimetrics Software).

**Novel Object Recognition.** An open box (45 x 45 x 35 cm) was used to conduct the experiment. A camera was mounted above the open field. Before the experiment, mice were handled every day for one week. Two days before the experiment, they were allowed to explore the open box for 10 minutes to get familiar with the apparatus. Each test contained three sessions. In the first session, mice were placed into the empty open field without objects for 10 minutes. In the second session, two identical objects were added. Mice were

then placed into the open field for another 10 minutes to explore the objects. In the third session, the object presented was a duplicate of the sample presented in the second session to avoid olfactory trails, and a different object was introduced in the third session. In a control experiment (normal 24-hour natural sleep), the interval between second and third session was 24 hours. In the sleep deprivation (SD) experiment, after the second session, the mice were sleep deprived for five hours and allowed to recover for 19 hours in their home cages and after recovery sleep, the third session was performed.

[1] Srinivas, S., Watanabe, T., Lin, C.S., William, C.M., Tanabe, Y., Jessell, T.M., and Costantini, F. (2001) Cre reporter strains produced by targeted insertion of EYFP and ECFP in to the ROSA26 locus. BMC Dev Biol *1*, 4
